# Supplementary material for: Cadaveric simulation versus standard training for postgraduate trauma and orthopaedic surgical trainees: protocol for the CAD:TRAUMA study multicentre randomised controlled educational trial
Source: BMJ Open. 2020 Sep 25;10(9):e037319. doi: 10.1136/bmjopen-2020-037319 (PMC7520841; doi:10.1136/bmjopen-2020-037319)
Supplement: Author's manuscript [file bmjopen-2020-037319.draft_revisions.pdf]

BMJ Open is committed to open peer review. As part of this commitment we make the peer review history of every article we publish publicly available.

When an article is published we post the peer reviewers' comments and the authors' responses online. We also post the versions of the paper that were used during peer review. These are the versions that the peer review comments apply to.

The versions of the paper that follow are the versions that were submitted during the peer review process. They are not the versions of record or the final published versions. They should not be cited or distributed as the published version of this manuscript.

BMJ Open is an open access journal and the full, final, typeset and author-corrected version of record of the manuscript is available on our site with no access controls, subscription charges or pay-per-view fees (http://bmjopen.bmj.com).

If you have any questions on BMJ Open's open peer review process please email info.bmjopen@bmj.com

BMJ Open

Cadaveric simulation vs standard training for postgraduate trauma & orthopaedic surgical trainees: protocol for the CAD:TRAUMA study multi-centre randomised controlled educational trial

Journal:	*BMJ Open*
Manuscript ID	bmjopen-2020-037319
Article Type:	Protocol
Date Submitted by the Author:	28-Jan-2020

Complete List of Authors:	James, Hannah; University of Warwick Warwick Medical School, Clinical Trials Unit; University Hospitals Coventry and Warwickshire NHS Trust, Trauma & Orthopaedic Surgery<br>Pattison, Giles; University Hospitals Coventry and Warwickshire NHS Trust, Trauma & Orthopaedic Surgery<br>Fisher, Joanne; University of Warwick Warwick Medical School, Clinical Trials Unit<br>Griffin, Damian; University of Warwick, Warwick Medical School

Keywords:	MEDICAL EDUCATION & TRAINING, ORTHOPAEDIC & TRAUMA SURGERY, Trauma management < ORTHOPAEDIC & TRAUMA SURGERY

SCHOLARONE™ Manuscripts

I, the Submitting Author has the right to grant and does grant on behalf of all authors of the Work (as defined in the below author licence), an exclusive licence and/or a non-exclusive licence for contributions from authors who are: i) UK Crown employees; ii) where BMJ has agreed a CC-BY licence shall apply, and/or iii) in accordance with the terms applicable for US Federal Government officers or employees acting as part of their official duties; on a worldwide, perpetual, irrevocable, royalty-free basis to BMJ Publishing Group Ltd ("BMJ") its licensees and where the relevant Journal is co-owned by BMJ to the co-owners of the Journal, to publish the Work in this journal and any other BMJ products and to exploit all rights, as set out in our licence.

The Submitting Author accepts and understands that any supply made under these terms is made by BMJ to the Submitting Author unless you are acting as an employee on behalf of your employer or a postgraduate student of an affiliated institution which is paying any applicable article publishing charge ("APC") for Open Access articles. Where the Submitting Author wishes to make the Work available on an Open Access basis (and intends to pay the relevant APC), the terms of reuse of such Open Access shall be governed by a Creative Commons licence – details of these licences and which Creative Commons licence will apply to this Work are set out in our licence referred to above.

Other than as permitted in any relevant BMJ Author's Self Archiving Policies, I confirm this Work has not been accepted for publication elsewhere, is not being considered for publication elsewhere and does not duplicate material already published. I confirm all authors consent to publication of this Work and authorise the granting of this licence.

For peer review only

Cadaveric simulation vs standard training for postgraduate trauma & orthopaedic surgical trainees: protocol for the CAD:TRAUMA study multi-centre randomised controlled educational trial

Hannah K James[1,2] h.smith.1@warwick.ac.uk

Giles T R Pattison[2] Giles.Pattison@uhcw.nhs.uk

Joanne D Fisher[1] Joanne.Fisher@warwick.ac.uk

Damian R Griffin[1,2] Damian.Griffin@warwick.ac.uk

1. Clinical Trials Unit, Warwick Medical School, Coventry, CV4 7HL
2. Department of Trauma & Orthopaedic Surgery, University Hospitals Coventry & Warwickshire, Clifford Bridge Road, Coventry, CV2 2DX

Correspondence:

Dr HK James

For peer review only

Clinical Sciences Research Laboratories

Clinical Sciences Building

University Hospital Coventry & Warwickshire

Clifford Bridge Road

Coventry

CV2 2DX

Tel 07786737479

Word Count 3,420

Keywords

Education, Training, Simulation

For peer review only

Abstract

Introduction

The quantity and quality of surgical training in the UK has been negatively affected by reduced working hours and NHS financial pressures. Traditionally surgical training has occurred by the master-apprentice model involving a process of graduated responsibility, but a modern alternative is to use simulation for the early stages of training. It is not known if simulation training for junior trainees can safeguard patients and improve clinical outcomes.

This paper details the protocol for a multicentre randomised controlled educational trial of a cadaveric simulation training intervention versus standard training for junior postgraduate orthopaedic surgeons-in-training. This is the first study to assess the effect of cadaveric simulation training on patient outcome.

Methods and Analysis

We will recruit postgraduate orthopaedic surgeons-in-training in the first three years (of eight) of the specialist training programme. Participants will be block randomised and allocated to either cadaveric simulation or standard 'on-the-job' training, learning three common orthopaedic procedures, each of which is a sub-study within the trial. The procedures are 1) Dynamic Hip Screw(DHS), 2) hemiarthroplasty and 3) ankle fracture fixation. These procedures have been selected as they are very common procedures which are routinely performed by junior surgeons-in-training. A pragmatic approach to sample size is taken in lieu of a formal power calculation as this is novel exploratory work with no a priori estimate of effect size to reference. The primary outcome measure is the technical success of the surgery performed on patients by the participating surgeons-in-training during the follow-up period for the three sub-study procedures, as measured by the implant position on the post-operative radiograph. The secondary outcome measures are procedure time, post-operative complication rate and patient health state at 4 months post-operation (EQ-5D – substudies 1 and 2 only).

Ethics, registration and dissemination

For peer review only

National research ethics approval was granted for this study (15/WM/0464). Confidentiality Advisory Group approval was granted for accessing radiographic and outcome data without patient consent on 27 February 2017 (16/CAG/0125). The results of this trial will be submitted to a peer-reviewed journal and will inform educational and clinical practice.

Trial registration number

ISRCTN20431944; Pre-results

Protocol version V_P_1.1

Article Summary

Strengths and limitations of this study:

- Surgeons-in-training should not be doing operations for the first time on living patients.
- Simulation using deceased human bodies may be a good way to train novice surgeons at the early stages of learning, until they are competent enough to operate on real patients.
- This is the first randomised trial assessing the impact of a cadaveric simulation intervention on patient outcomes

For peer review only

- We cannot do a power calculation as this is novel work and we do not know what the effect size is likely to be

- Sample size is limited by the capacity of the surgical training centre

For peer review only

Introduction

It is imperative that surgeons are trained to a high standard, so they can perform safe and effective operations for patients. The quality and quantity of surgical training in the UK is currently under threat from a 'perfect storm' of factors(1). These include reduced working hours(2, 3), shift based working patterns(4) with the loss of the traditional surgical firm, and a move to expedite training and shorten specialist programmes(5, 6). This is set within a climate of unprecedented financial austerity in the NHS and ever increasing service pressures.

Simulation offers a solution to some of these challenges by moving the early part of the surgical learning curve away from patients into a controlled environment, where skills can be rapidly acquired and competency can be assessed before trainees are released into clinical practice(7). Simulation is also potentially a very efficient way of training, as large numbers of

trainees can be trained simultaneously, at an intensity not feasible in the clinical environment due to competing service demands.

Cadaveric simulation - training using deceased, preserved or fresh human bodies - is a particularly promising modality for training. It offers a highly realistic 'high fidelity' simulation where surgical anatomy is represented as in life, including soft tissues and neurovascular relationships(8, 9). This allows for a much more realistic simulated operation than would be possible on a plastic model or virtual reality simulator. In addition to the superior anatomic and tactile (haptic) properties of cadavers, the whole operating theatre environment can be simulated, including (but not limited to) surgical dress, draping, instrumentation and multidisciplinary team. This 'whole dress rehearsal' for surgery may enhance development of non-technical skills in addition to the technical operative surgical skills(10).

There is abundant low quality evidence showing cadaveric simulation may induce short term skill improvement as measured by subjective and behavioural metrics, but there is a lack of high quality, quantitative evidence that skills learnt in cadaveric simulation can transfer to the workplace, leading to improved outcomes for patients(8).

Our trial attempts to address this evidence deficit, and is both topical and timely.

Good Clinical Practice

For peer review only

This trial will be undertaken in compliance with Good Practice Guidelines, complying with the Declaration of Helsinki and UK Legislation. Warwick standard operating procedures (SOPs) will be followed.

Consolidated Standards of Reporting Trials

The results of the trial will be reported in line with the Consolidated Standard of Reporting (CONSORT) statement(11). This protocol has been written according to the SPIRIT reporting guidelines(12).

Aim

The aim is to determine which of the two surgical training strategies for junior orthopaedic surgeons-in-training leads to the best patient outcomes for three common procedures.

Objectives

1. To assess the impact of a cadaveric simulation training intervention on the patient outcome of operations performed by junior orthopaedic surgeons-in-training
2. To define the early learning curve of DHS, Hemiarthroplasty and ankle fracture fixation
3. To explore the feasibility of using post-operative x-rays to assess technical skill

For peer review only

Methods and Analysis

Study design

This is a UK multicentre, two-arm, group parallel randomised controlled educational trial

Sample size

This trial is the first attempt to objectively measure transfer of open operative skills from cadaveric simulation into the workplace using patient-based outcome measures. There is no available estimate of effect size to reference against a priori in determining sample size, therefore a pragmatic approach to sample size will be taken in lieu of a formal power calculation. The surgical training centre can accommodate 16 delegates at one time and financial resources permitted one iteration of the cadaveric training course. Our maximum sample size is therefore 16 participants in each arm of the study

Outcome measures

A) Radiographic outcomes

For peer review only

The radiographs will be obtained electronically from hospital servers and the implant position measured manually using computer software. The operations will be identified retrospectively by access to the participating surgeons' electronic logbooks. The measurements vary by operation type and are defined as follows.

Sub-study 1: Dynamic Hip Screw

1. Primary Outcome
   - Tip-Apex distance (in mm)
2. Secondary Outcomes (in order of importance)
   - Lag screw position in the femoral head (defined by Cleveland Zones)
   - Plate flush to lateral femoral cortex (binary Y/N)
   - 8 cortex hold for plate screws (binary Y/N)

Sub- study 2: Hemiarthroplasty

1. Primary Outcome
   - Leg length discrepancy (mm)
2. Secondary Outcomes (in order of importance)
   - Femoral stem alignment (degrees off neutral)
   - Cement mantle quality (Barrack grade score)
   - Femoral offset change relative to native hip (mm)

Sub- study 3: Ankle fracture fixation

For peer review only

1. Primary Outcome

   - Medial clear space (mm)

2. Secondary Outcomes

   - Lateral malleolar displacement (mm)
   - Tibiofibular clear space (mm)
   - Talocrural angle (degrees)
   - Medial malleolar displacement (mm)

B) Clinical Outcomes

The clinical outcome measures for sub-studies 1-3 are;

1) Procedure Time

Defined as knife-to-skin/surgical start time to wound closure/surgical stop time. These will be obtained from hospital theatre management systems. Procedure time has been chosen as an outcome measure as there is evidence in the literature that procedure time is inversely related to experience, and so can be used as a surrogate measure of technical proficiency(13)

2) Intra-operative radiation dose to patient

For peer review only

Defined as time under fluoroscopy (seconds) and radiation dose (mGym2). There is evidence that with increasing experience and skill, surgeons use less intra-operative x-rays to adjust the position of the fracture and implant(13). Hemiarthroplasty does not require fluoroscopy so this will not be used as an outcome measure for sub-study 2.

3) Post-operative complication rate

The complications of interest are the acute post-operative complications during the inpatient admission. These will be sub-categorised as acute medical complications (hospital acquired pneumonia, renal complications, cardiac complications, DVT/PE) and surgical complications (wound infection, wound dehiscence, metalwork failure, deep infection).

4) Health state at 4 months post-operation (EQ-5D)

Health state at 4-months post-operation will be measured using EQ-5D, which is a standardized instrument measuring generic health status, which has been widely validated in clinical trials. This data is being collected separately as part of the WHiTE comprehensive cohort study of hip fracture patients (ISRCTN63982700) and reported elsewhere(14). EQ-5D will be used for sub-studies 1 and 2 only as these involve hip fractures.

Screening and eligibility

For peer review only

Orthopaedic surgeons-in-training in their first, second or third specialist training year in the West Midlands Workforce Deanery will be eligible to participate in the trial. Eligible trainees will be identified by liaison with the training programme directors for Trauma & Orthopaedic surgery in the three West Midlands schools; Warwick, Birmingham and Oswestry. An invitation email will be sent to all eligible trainees by programme administrators at the deanery.

Inclusion criteria

1) Trauma & Orthopaedic surgeon-in-training in West Midlands school (Warwick/Birmingham/Oswestry)
2) In specialty training year 1-3
3) Willing and able to attend a two-day cadaveric simulation training course at the West Midlands Surgical Training Centre, Coventry

Exclusion criteria

1) Unavailable on course dates

Consent

Surgeon participants:

For peer review only

Potential study participants will be provided with written and verbal information about the study. Consent will be obtained by the trial team. The right to refuse participation without giving reasons will be fully respected, and enrolled participants will be free to withdraw from the study at any time without reason, and without prejudice to their training. All participants will be provided with the contact information of a team member who can provide further information about the study. All participants who are allocated to the control group will have the opportunity to undertake the cadaveric simulation training intervention at the end of the study follow-up. This provision is being offered so that the control group are not disadvantaged in their access to educational opportunity by virtue of being randomised to the control group.

Patients whose operations are assessed:

Patients who undergo an operation by a surgeon who is participating in the study will not be separately consented to allow access to radiographs to assess their implant position or clinical outcome data. Permission to access this information for the purposes of this study without patient consent has been granted from the confidentiality advisory group (16/CAG/0125). It is recognised that seeking consent from a group of primarily elderly, frail patients to assess low risk, routine clinical data in a secure manner for a trial they are not directly participating in would be unduly burdensome for the patients. All patient data will be fully anonymised and handled securely in line with university data regulations.

Randomisation

For peer review only

Participants will be randomised at the point of recruitment using block randomisation (block size 4) to generate a random sequence list, to which participants will be assigned in the order that they enter the study. The allocation sequence will be generated by a senior medical statistician, participants will be enrolled by the trial team.

Postrandomisation withdrawals

Withdrawn participants will not be replaced.

Study setting

The study participants will be on training rotations within the regional hospitals of the West Midlands during the study follow-up. The hospitals where trainees have been working, and performing operations, during the study follow up will be identified from the participants electronic surgical logbook records.

Interventions

Control group

For peer review only

The control group will undertake normal clinical training ‘on-the-job’ according to the master-apprentice model, which is the current standard training practice.

Intervention group (cadaveric simulation trained)

Participants allocated to the intervention group will receive an intensive, 2-day cadaveric simulation training course at the start of the training year, where 4 common orthopaedic surgical procedures will be taught (DHS, hemiarthroplasty, ankle fracture fixation and lower limb fasciotomy). All intervention participants will receive training on all 4 procedures, which will be considered separately in the analysis as individual sub-studies (as they have different radiographic outcome measures). The fasciotomy procedure is included as a ‘filler’ to make the course structure work, and chosen because it is an important high-stakes, anatomically critical operation that is rarely performed by trainees. Outcomes related to the fasciotomy procedure will not be collected or included in the analysis.

The cadaveric simulation training course

The course will be delivered in September at the start of the surgical training year (which runs August to August). The course will take place at the West Midlands Surgical Training Centre (WMSTC) at the University Hospital Coventry & Warwickshire (UHCW). The WMSTC is a specialized wet-laboratory facility for delivering cadaveric training, and has an experienced dedicated faculty to facilitate training delivery.

For peer review only

The course will consist of two full days of teaching, with expert consultant faculty teaching on fresh-frozen hemi-cadavers (waist-to-toe-tip). The participant:faculty ratio will be 2:1, and participant:cadaver ratio will be 2:1. Each participant will undertake each of the four procedures in their entirety as primary surgeon('skin-to-skin'), and will act as assistant when their partner is the primary surgeon four times. Hence is participant is exposed to eight procedures during the course:

The environment and psychological fidelity of the simulation will be maximised by providing;

- Full surgical dress including masks, gloves, gowns and lead x-ray aprons
- The usual disposable surgical drapes
- Skin preparation (iodine solution) to prepare the surgical site, and participants and faculty will be asked to observe the usual sterile field precautions as in real theatre
- Full surgical instrument trays, surgical implants and cement (for hemiarthroplasty) of the same type as in real theatre will be used
- Image intensifier (mobile x-ray) will be available for intra-operative use
- Background noise levels and room temperature were maintained at what would usually be expected in the operating theatre

The simulated operating theatres will be set up within the WMSTC as two parallel round-robin circuits. The two stations requiring x-ray use (DHS and ankle ORIF) will be set up at the far end of the room to create a radiation zone and where appropriate, standard precautions will be used. Careful consideration will be given to the optimum sequential use

For peer review only

of the cadaveric specimens in planning the course structure. For example, it is necessary that the DHS station precedes the hemiarthroplasty station as it would obviously not be possible to perform a DHS operation when the femoral head had been removed. Similarly, the fasciotomy incisions would compromise the soft tissue envelope of the lower limb to a sufficient degree that the fidelity of the ankle ORIF station would be compromised. It is important to make the best and most efficient use of the cadaveric material, for both ethical and financial reasons.

For peer review only

Blinding

The participants cannot be blinded to the type of training they receive, neither can the trial team in organising the cadaveric simulation training. The trial team will take no part in the training of participants. The assessment of radiographic images will be made blinded to group allocation.

Adverse event management

In the unlikely event of a serious adverse event (SAE), the chief investigator will report to the sponsor (University of Warwick), ethics committee and project supervisors.

Patient and public involvement

There was no direct patient or public involvement in the design of the study, although clearly training competent surgeons is in the public interest. There is qualitative work to be done around this trial to better understand patient expectations of surgical training.

End of trial

The trial will end when data collection is complete. The trial will be stopped prematurely if required by the ethics committee, following recommendations from the sponsor, or if funding for the study is withdrawn. The research ethics committee and confidentiality advisory group will be notified in writing once the trial is complete.

Trial Oversight

This trial is being undertaken as part of a doctoral research project (HKJ), and supervised by three senior supervisors (DG, JF, GTRP). The supervisors will act as the trial management group and steering committee. The trial is being conducted within a registered Clinical Trials Unit, and will follow the CTU standard operating procedures.

Data Collection Plan

The operations performed by the participants will be identified by the surgeons' electronic logbook. These will be extracted and anonymised to study identifier by the electronic logbook data team, before being sent to the trial team. The data will include operation type, date, hospital, hospital ID, patient age, American Society of Anaesthesiologists Grade and

For peer review only

supervision code. The radiographs and clinical outcome data relating to these procedures will then be obtained from the study sites via liaison with the respective Research & Development Departments. Data will be entered into a secure trial database on a professionally encrypted trial-specific computer, fully anonymised with only study identifiers. Once data collection is complete, and prior to analysis, range checks for data values will be undertaken, and data will be double checked on entry to the statistical software package. The project supervisors will act as the data monitoring committee. No interim analysis will be undertaken. The trial team and statistician will have access to the final trial dataset.

Statistical Analysis Plan

Baseline data will be summarised and compared between the two arms of the study. A CONSORT chart showing the flow of participants through the study will be produced. The three taught procedures (substudies 1-3) will be analysed and reported individually.

The main analysis will investigate and report differences between the two groups with respect to the implant positions (as measured from radiographs), the procedure times, the intra-operative radiation dose to the patient, and patient outcomes, as measured by post-operative complications and health state at 4 months post-operation (hip fractures only).

Statistical tests will be two-sided and considered to demonstrate a significant difference when $p<0.05$. Temporal trends by group for implant position, procedure time and radiation dose will be presented. Linear mixed effects models will be fitted to allow for within-surgeon correlation between repeated observations (surgeon clustering as a random effect), and to

For peer review only

adjust for important co-variates such as patient condition, age and surgeon experience. These will be summarised by plotting individual learning curves, and then modelled to estimate the overall learning curves for the two arms of the study.

Descriptive statistical analyses of between-group comparisons will be presented for complication rate and health state, with temporal analysis of the latter being reported if appropriate and feasible. The statistical analysis will be supervised and checked by a senior medical statistician at Warwick University.

In the event of missing data, statistician advice will be sought on multiple imputation.

Ethics and dissemination

Master-apprentice 'on-the-job' training for surgeons is the current training standard in the UK(15, 16), and therefore the control arm of the study reflects usual practice. The cadaveric simulation training intervention is a novel experimental intervention, but as it is an educational intervention it does not expose trial participants to any substantial risks of harm. The trial results will be reported in accordance with the CONSORT statement, and disseminated through publication in peer reviewed journals and conferences. The results of the trial will be presented to Health Education England and the Royal Surgical College. The dataset, statistical code and technical appendices will be made available on request to the corresponding author.

Funding

For peer review only

This work was supported by Versus Arthritis grant number 20845

Sponsor Contact Information

Mrs Jane Prewitt, University of Warwick, Research & Impact Services, University House, Kirby Corner Road, Coventry, CV4 8UW

wmssponsorship@warwick.ac.uk

Declaration/Conflict of Interests

None to declare

References:

1. Temple J. Time for Training. A Review of the impact of the European Working Time Directive on the quality of training. London: Medical Education England; 2010.
2. Maxwell AJ, Crocker M, Jones TL, Bhagawati D, Papadopoulos MC, Bell BA. Implementation of the European Working Time Directive in neurosurgery reduces continuity of care and training opportunities. Acta Neurochirurgica. 2010;152(7):1207-10.
3. Pickersgill T. The European working time directive for doctors in training. BMJ. 2001;323(7324):1266.
4. Marron C, Byrnes C, Kirk S. An EWTD-compliant shift rota decreases training opportunities. Bulletin of the Royal College of Surgeons of England. 2005;87(7):246-8.
5. Shape of Training. Report from the UK Shape of Training Steering Group (UKSTSG). General Medical Council; 2017.
6. Royal College of Surgeons of England. Improving Surgical Training - Trainee Prospectus. London: The Royal College of Surgeons; 2018.
7. Curran I. A Framework for Technology Enhanced Learning. London: Department of Health; 2011.
8. James H, Chapman A, Pattison G, Griffin DR, Fisher J. Current status of Cadaveric Simulation for surgical training: a Systematic Review. British Journal of Surgery. 2019.
9. Gilbody J, Prasthofer A, Ho K, Costa M. The use and effectiveness of cadaveric workshops in higher surgical training: a systematic review. Annals of the Royal College of Surgeons of England. 2011;93(5):347-56.
10. James H, Pattison G, Griffin D, Fisher J. How does cadaveric simulation influence learning in Orthopedic residents? Journal of Surgical Education. 2020;In Press.
11. Schulz KF, Altman DG, Moher D. CONSORT 2010 Statement: updated guidelines for reporting parallel group randomised trials. BMJ. 2010;340.
12. Chan A-W, Tetzlaff JM, Altman DG, Laupacis A, Gøtzsche PC, Krleža-Jerić K, et al. SPIRIT 2013 Statement: Defining Standard Protocol Items for Clinical Trials. Annals of Internal Medicine. 2013;158(3):200-7.
13. James H, Chapman A, Pattison G, Fisher JG, Griffin DR. Assessment of technical skill acquisition and operative competence in Trauma and Orthopaedic surgical training: A systematic review. JBJS(Am) Reviews. 2019.
14. Costa ML, Griffin XL, Achten J, Metcalfe D, Judge A, Pinedo-Villanueva R, et al. World Hip Trauma Evaluation (WHiTE): framework for embedded comprehensive cohort studies. BMJ Open. 2016;6(10):e011679.
15. Reznick RK, MacRae H. Teaching surgical skills--changes in the wind. N Engl J Med. 2006;355(25):2664-9.

For peer review only

16. Hargreaves DH, Bowditch MG, Griffin DR. On-the-job training for surgeons. 1 ed. London: The Royal Society of Medicine Press; 1997.

Author Statement/Contributions

HKJ designed the study and wrote the manuscript

GTRP co-designed the study and the intervention and edited the manuscript

JDF edited the manuscript, made a substantial contribution to the design and is lead supervisor for the qualitative part of the project

DG co-designed the study, edited draft protocols and is lead supervisor for the quantitative part of the project

Acknowledgements

None

For peer review only

Reporting checklist for protocol of a clinical trial.

Based on the SPIRIT guidelines.

Instructions to authors

Complete this checklist by entering the page numbers from your manuscript where readers will find each of the items listed below.

Your article may not currently address all the items on the checklist. Please modify your text to include the missing information. If you are certain that an item does not apply, please write "n/a" and provide a short explanation.

Upload your completed checklist as an extra file when you submit to a journal.

In your methods section, say that you used the SPIRITreporting guidelines, and cite them as:

Chan A-W, Tetzlaff JM, Altman DG, Laupacis A, Gøtzsche PC, Krleža-Jerić K, Hróbjartsson A, Mann H, Dickersin K, Berlin J, Doré C, Parulekar W, Summerskill W, Groves T, Schulz K, Sox H, Rockhold FW, Rennie D, Moher D. SPIRIT 2013 Statement: Defining standard protocol items for clinical trials. Ann Intern Med. 2013;158(3):200-207

		Reporting Item	Page Number
**Administrative information**			
Title	#1	Descriptive title identifying the study design, population, interventions, and, if applicable, trial acronym	1
Trial registration	#2a	Trial identifier and registry name. If not yet registered, name of intended registry	3
Trial registration: data set	#2b	All items from the World Health Organization Trial Registration Data Set	3
Protocol version	#3	Date and version identifier	3
Funding	#4	Sources and types of financial, material, and other support	15
Roles and responsibilities: contributorship	#5a	Names, affiliations, and roles of protocol contributors	1

For peer review only

Roles and responsibilities: sponsor contact information	#5b	Name and contact information for the trial sponsor	15
Roles and responsibilities: sponsor and funder	#5c	Role of study sponsor and funders, if any, in study design; collection, management, analysis, and interpretation of data; writing of the report; and the decision to submit the report for publication, including whether they will have ultimate authority over any of these activities	14
Roles and responsibilities: committees	#5d	Composition, roles, and responsibilities of the coordinating centre, steering committee, endpoint adjudication committee, data management team, and other individuals or groups overseeing the trial, if applicable (see Item 21a for data monitoring committee)	14
**Introduction**			
Background and rationale	#6a	Description of research question and justification for undertaking the trial, including summary of relevant studies (published and unpublished) examining benefits and harms for each intervention	1
Background and rationale: choice of comparators	#6b	Explanation for choice of comparators	11
Objectives	#7	Specific objectives or hypotheses	5-6
Trial design	#8	Description of trial design including type of trial (eg, parallel group, crossover, factorial, single group), allocation ratio, and framework (eg, superiority, equivalence, non-inferiority, exploratory)	5
**Methods: Participants, interventions, and outcomes**			
Study setting	#9	Description of study settings (eg, community clinic, academic hospital) and list of countries where data will be collected. Reference to where list of study sites can be obtained	10

For peer review only

Eligibility criteria	#10	Inclusion and exclusion criteria for participants. If applicable, eligibility criteria for study centres and individuals who will perform the interventions (eg, surgeons, psychotherapists)	9
Interventions: description	#11a	Interventions for each group with sufficient detail to allow replication, including how and when they will be administered	11-13
Interventions: modifications	#11b	Criteria for discontinuing or modifying allocated interventions for a given trial participant (eg, drug dose change in response to harms, participant request, or improving / worsening disease)	N/A – educational trial
Interventions: adherance	#11c	Strategies to improve adherence to intervention protocols, and any procedures for monitoring adherence (eg, drug tablet return; laboratory tests)	N/A – educational trial
Interventions: concomitant care	#11d	Relevant concomitant care and interventions that are permitted or prohibited during the trial	N/A – educational trial
Outcomes	#12	Primary, secondary, and other outcomes, including the specific measurement variable (eg, systolic blood pressure), analysis metric (eg, change from baseline, final value, time to event), method of aggregation (eg, median, proportion), and time point for each outcome. Explanation of the clinical relevance of chosen efficacy and harm outcomes is strongly recommended	6-7
Participant timeline	#13	Time schedule of enrolment, interventions (including any run-ins and washouts), assessments, and visits for participants. A schematic diagram is highly recommended (see Figure)	11
Sample size	#14	Estimated number of participants needed to achieve study objectives and how it was determined, including clinical and statistical assumptions supporting any sample size calculations	6
Recruitment	#15	Strategies for achieving adequate participant enrolment to reach target sample size	8

For peer review only

**Methods: Assignment of interventions (for controlled trials)**			
Allocation: sequence generation	#16a	Method of generating the allocation sequence (eg, computer-generated random numbers), and list of any factors for stratification. To reduce predictability of a random sequence, details of any planned restriction (eg, blocking) should be provided in a separate document that is unavailable to those who enrol participants or assign interventions	10
Allocation concealment mechanism	#16b	Mechanism of implementing the allocation sequence (eg, central telephone; sequentially numbered, opaque, sealed envelopes), describing any steps to conceal the sequence until interventions are assigned	10
Allocation: implementation	#16c	Who will generate the allocation sequence, who will enrol participants, and who will assign participants to interventions	10
Blinding (masking)	#17a	Who will be blinded after assignment to interventions (eg, trial participants, care providers, outcome assessors, data analysts), and how	13
Blinding (masking): emergency unblinding	#17b	If blinded, circumstances under which unblinding is permissible, and procedure for revealing a participant's allocated intervention during the trial	N/A - educational trial
**Methods: Data collection, management, and analysis**			
Data collection plan	#18a	Plans for assessment and collection of outcome, baseline, and other trial data, including any related processes to promote data quality (eg, duplicate measurements, training of assessors) and a description of study instruments (eg, questionnaires, laboratory tests) along with their reliability and validity, if known. Reference to where data collection forms can be found, if not in the protocol	14
Data collection plan: retention	#18b	Plans to promote participant retention and complete follow-up, including list of any outcome data to be collected for	14

For peer review only

		participants who discontinue or deviate from intervention protocols	
Data management	#19	Plans for data entry, coding, security, and storage, including any related processes to promote data quality (eg, double data entry; range checks for data values). Reference to where details of data management procedures can be found, if not in the protocol	14
Statistics: outcomes	#20a	Statistical methods for analysing primary and secondary outcomes. Reference to where other details of the statistical analysis plan can be found, if not in the protocol	14-15
Statistics: additional analyses	#20b	Methods for any additional analyses (eg, subgroup and adjusted analyses)	14-15
Statistics: analysis population and missing data	#20c	Definition of analysis population relating to protocol non-adherence (eg, as randomised analysis), and any statistical methods to handle missing data (eg, multiple imputation)	15
**Methods: Monitoring**			
Data monitoring: formal committee	#21a	Composition of data monitoring committee (DMC); summary of its role and reporting structure; statement of whether it is independent from the sponsor and competing interests; and reference to where further details about its charter can be found, if not in the protocol. Alternatively, an explanation of why a DMC is not needed	14
Data monitoring: interim analysis	#21b	Description of any interim analyses and stopping guidelines, including who will have access to these interim results and make the final decision to terminate the trial	14
Harms	#22	Plans for collecting, assessing, reporting, and managing solicited and spontaneously reported adverse events and other unintended effects of trial interventions or trial conduct	N/A – educational trial
Auditing	#23	Frequency and procedures for auditing trial conduct, if any, and whether the process will be independent from investigators and the sponsor	N/A – educational trial
**Ethics and dissemination**			

For peer review only

Research ethics approval	#24	Plans for seeking research ethics committee / institutional review board (REC / IRB) approval	3
Protocol amendments	#25	Plans for communicating important protocol modifications (eg, changes to eligibility criteria, outcomes, analyses) to relevant parties (eg, investigators, REC / IRBs, trial participants, trial registries, journals, regulators)	N/A – educational trial
Consent or assent	#26a	Who will obtain informed consent or assent from potential trial participants or authorised surrogates, and how (see Item 32)	9
Consent or assent: ancillary studies	#26b	Additional consent provisions for collection and use of participant data and biological specimens in ancillary studies, if applicable	N/A – educational trial
Confidentiality	#27	How personal information about potential and enrolled participants will be collected, shared, and maintained in order to protect confidentiality before, during, and after the trial	14
Declaration of interests	#28	Financial and other competing interests for principal investigators for the overall trial and each study site	16
Data access	#29	Statement of who will have access to the final trial dataset, and disclosure of contractual agreements that limit such access for investigators	14
Ancillary and post trial care	#30	Provisions, if any, for ancillary and post-trial care, and for compensation to those who suffer harm from trial participation	N/A – educational trial
Dissemination policy: trial results	#31a	Plans for investigators and sponsor to communicate trial results to participants, healthcare professionals, the public, and other relevant groups (eg, via publication, reporting in results databases, or other data sharing arrangements), including any publication restrictions	15
Dissemination policy: authorship	#31b	Authorship eligibility guidelines and any intended use of professional writers	15
Dissemination policy: reproducible research	#31c	Plans, if any, for granting public access to the full protocol, participant-level dataset, and statistical code	N/A

Appendices

For peer review only

Informed consent materials	#32	Model consent form and other related documentation given to participants and authorised surrogates	Appendix
Biological specimens	#33	Plans for collection, laboratory evaluation, and storage of biological specimens for genetic or molecular analysis in the current trial and for future use in ancillary studies, if applicable	N/A – educational trial

None The SPIRIT checklist is distributed under the terms of the Creative Commons Attribution License CC-BY-ND 3.0. This checklist can be completed online using https://www.goodreports.org/, a tool made by the EQUATOR Network in collaboration with Penelope.ai

For peer review only

BMJ Open

Cadaveric simulation vs standard training for postgraduate trauma & orthopaedic surgical trainees: protocol for the CAD:TRAUMA study multi-centre randomised controlled educational trial

Journal:	*BMJ Open*
Manuscript ID	bmjopen-2020-037319.R1
Article Type:	Protocol
Date Submitted by the Author:	04-May-2020
Complete List of Authors:	James, Hannah; University of Warwick Warwick Medical School, Clinical Trials Unit; University Hospitals Coventry and Warwickshire NHS Trust, Trauma & Orthopaedic Surgery<br>Pattison, Giles; University Hospitals Coventry and Warwickshire NHS Trust, Trauma & Orthopaedic Surgery<br>Fisher, Joanne; University of Warwick Warwick Medical School, Clinical Trials Unit<br>Griffin, Damian; University of Warwick, Warwick Medical School
**Primary Subject Heading**:	Medical education and training
Secondary Subject Heading:	Surgery
Keywords:	MEDICAL EDUCATION & TRAINING, ORTHOPAEDIC & TRAUMA SURGERY, Trauma management < ORTHOPAEDIC & TRAUMA SURGERY

SCHOLARONE™ Manuscripts

I, the Submitting Author has the right to grant and does grant on behalf of all authors of the Work (as defined in the below author licence), an exclusive licence and/or a non-exclusive licence for contributions from authors who are: i) UK Crown employees; ii) where BMJ has agreed a CC-BY licence shall apply, and/or iii) in accordance with the terms applicable for US Federal Government officers or employees acting as part of their official duties; on a worldwide, perpetual, irrevocable, royalty-free basis to BMJ Publishing Group Ltd ("BMJ") its licensees and where the relevant Journal is co-owned by BMJ to the co-owners of the Journal, to publish the Work in this journal and any other BMJ products and to exploit all rights, as set out in our licence.

The Submitting Author accepts and understands that any supply made under these terms is made by BMJ to the Submitting Author unless you are acting as an employee on behalf of your employer or a postgraduate student of an affiliated institution which is paying any applicable article publishing charge ("APC") for Open Access articles. Where the Submitting Author wishes to make the Work available on an Open Access basis (and intends to pay the relevant APC), the terms of reuse of such Open Access shall be governed by a Creative Commons licence – details of these licences and which Creative Commons licence will apply to this Work are set out in our licence referred to above.

Other than as permitted in any relevant BMJ Author's Self Archiving Policies, I confirm this Work has not been accepted for publication elsewhere, is not being considered for publication elsewhere and does not duplicate material already published. I confirm all authors consent to publication of this Work and authorise the granting of this licence.

For peer review only

Cadaveric simulation vs standard training for postgraduate trauma & orthopaedic surgical trainees: protocol for the CAD:TRAUMA study multi-centre randomised controlled educational trial

For peer review only

Hannah K James[1,2] h.smith.1@warwick.ac.uk

Giles T R Pattison[2] Giles.Pattison@uhcw.nhs.uk

Joanne D Fisher[1] Joanne.Fisher@warwick.ac.uk

Damian R Griffin[1,2] Damian.Griffin@warwick.ac.uk

1. Clinical Trials Unit, Warwick Medical School, Coventry, CV4 7HL
2. Department of Trauma & Orthopaedic Surgery, University Hospitals Coventry & Warwickshire, Clifford Bridge Road, Coventry, CV2 2DX

Correspondence:

Dr HK James

Clinical Sciences Research Laboratories

Clinical Sciences Building

University Hospital Coventry & Warwickshire

Clifford Bridge Road

Coventry

CV2 2DX

Tel 07786737479

Word Count 3,420

Keywords

Education, Training, Simulation

For peer review only

Abstract

Introduction

The quantity and quality of surgical training in the UK has been negatively affected by reduced working hours and NHS financial pressures. Traditionally surgical training has occurred by the master-apprentice model involving a process of graduated responsibility, but a modern alternative is to use simulation for the early stages of training. It is not known if simulation training for junior trainees can safeguard patients and improve clinical outcomes.

This paper details the protocol for a multicentre randomised controlled educational trial of a cadaveric simulation training intervention versus standard training for junior postgraduate orthopaedic surgeons-in-training. This is the first study to assess the effect of cadaveric simulation training for open surgery on patient outcome. The feasibility of delivering cadaveric training, use of radiographic and clinical outcome measures to assess impact and the challenges of upscaling provision will be explored.

Methods and Analysis

We will recruit postgraduate orthopaedic surgeons-in-training in the first three years (of eight) of the specialist training programme. Participants will be block randomised and allocated to either cadaveric simulation or standard 'on-the-job' training, learning three common orthopaedic procedures, each of which is a sub-study within the trial. The procedures are 1) Dynamic Hip Screw(DHS), 2) hemiarthroplasty and 3) ankle fracture fixation. These procedures have been selected as they are very common procedures which are routinely performed by junior surgeons-in-training. A pragmatic approach to sample size is taken in lieu of a formal power calculation as this is novel exploratory work with no a priori estimate of effect size to reference. The primary outcome measure is the technical success of the surgery performed on patients by the participating surgeons-in-training during the follow-up period for the three sub-study procedures, as measured by the implant position on the post-operative radiograph. The secondary outcome measures are procedure time, post-operative complication rate and patient health state at 4 months post-operation (EQ-5D – substudies 1 and 2 only).

For peer review only

Ethics, registration and dissemination

National research ethics approval was granted for this study by the NHS Research Authority South Birmingham Research Ethics Committee (15/WM/0464). Confidentiality Advisory Group approval was granted for accessing radiographic and outcome data without patient consent on 27 February 2017 (16/CAG/0125). The results of this trial will be submitted to a peer-reviewed journal and will inform educational and clinical practice.

For peer review only

Trial registration number

ISRCTN20431944; Pre-results

Protocol version V_P_1.1

<u>Article Summary</u>

Strengths and limitations of this study:

- This is the first randomised controlled trial assessing the impact of cadaveric simulation training on clinical outcomes
- Patient-centred outcome measures are used to measure an educational intervention for surgeons

- Multicentre study to maximize external validity of the results
- The training dose is small as cadaveric training is expense to deliver
- Pragmatic approach to sample size which is limited by the capacity of the surgical training centre

For peer review only

Introduction

It is imperative that surgeons are trained to a high standard, so they can perform safe and effective operations for patients. The quality and quantity of surgical training in the UK is currently under threat from a 'perfect storm' of factors(1). These include reduced working hours(2, 3), shift based working patterns(4) with the loss of the traditional surgical firm, and a move to expedite training and shorten specialist programmes(5, 6). This is set within a climate of unprecedented financial austerity in the NHS and ever increasing service pressures.

Simulation offers a solution to some of these challenges by moving the early part of the surgical learning curve away from patients into a controlled environment(7), where skills may be more rapidly acquired as compared to the clinical environment. Simulation is also

potentially a very efficient way of training, as large numbers of trainees can be trained simultaneously, at an intensity not feasible in the clinical environment due to competing service demands.

Cadaveric simulation - training using deceased, preserved or fresh human bodies - is a particularly promising modality for training. Fresh-frozen cadavers retain many of the soft tissue handling characteristics seen in live patients, and in combination with presenting the correct anatomy, particularly complex neurovascular relationships, may offer a more realistic simulated operation than would be possible on a plastic model or virtual reality simulator(8, 9). Cadaveric material does not bleed(10) and hence may be less useful for simulating procedures where haemorrhage control is an important feature.

The operating theatre environment can be simulated, including (but not limited to) surgical dress, draping, instrumentation and multidisciplinary team. This 'whole dress rehearsal' for surgery may enhance development of non-technical skills in addition to the technical operative surgical skills(11).

There are several challenges in delivering cadaveric simulation training. It is expensive to provide(9), particularly when cadaveric material has to be purchased under license where there is not a local body donation programme. It requires considerable infrastructure to deliver, including specialist wet laboratory facilities with the appropriately trained staff. These challenges become particularly pressing when provision of cadaveric training on a large scale is considered, and are an important driver in the development of high quality evidence

For peer review only

of educational impact. This evidence is necessary before considerable financial investment can be recommended in providing cadaveric simulation training on a larger scale.

There is abundant low quality evidence showing cadaveric simulation may induce short term skill improvement as measured by subjective and behavioural metrics, but there is a lack of high quality, quantitative evidence that skills learnt in cadaveric simulation can transfer to the workplace, leading to improved outcomes for patients(8).

Our trial attempts to address this evidence deficit, and is both topical and timely.

<u>Good Clinical Practice</u>

This trial will be undertaken in compliance with Good Practice Guidelines, complying with the Declaration of Helsinki and UK Legislation. Warwick standard operating procedures (SOPs) will be followed.

<u>Consolidated Standards of Reporting Trials</u>

The results of the trial will be reported in line with the Consolidated Standard of Reporting (CONSORT) statement(12). This protocol has been written according to the SPIRIT reporting guidelines(13).

<u>Aim</u>

For peer review only

The aim is to determine which of the two surgical training strategies for junior orthopaedic surgeons-in-training leads to the best patient outcomes for three common procedures.

Objectives

1. To assess the impact of a cadaveric simulation training intervention on the patient outcome of operations performed by junior orthopaedic surgeons-in-training
2. To define the early learning curve of DHS, Hemiarthroplasty and ankle fracture fixation
3. To explore the feasibility of using post-operative x-rays to assess technical skill

Methods and Analysis

Study design

This is a UK multicentre, two-arm, group parallel randomised controlled educational trial

Sample size

For peer review only

This trial is the first attempt to objectively measure transfer of open operative skills from cadaveric simulation into the workplace using patient-based outcome measures. There is no available estimate of effect size to reference against a priori in determining sample size, therefore a pragmatic approach to sample size will be taken in lieu of a formal power calculation. The surgical training centre can accommodate 16 delegates at one time and financial resources permitted one iteration of the cadaveric training course. Our maximum sample size is therefore 16 participants in each arm of the study

For peer review only

Outcome measures

A) Radiographic outcomes

The radiographs will be obtained electronically from hospital servers and the implant position measured manually using computer software. The operations will be identified retrospectively by access to the participating surgeons' electronic logbooks. The measurements vary by operation type and are defined as follows.

Sub-study 1: Dynamic Hip Screw

1. Primary Outcome
   - Tip-Apex distance (in mm)
2. Secondary Outcomes (in order of importance)
   - Lag screw position in the femoral head (defined by Cleveland Zones)

- Plate flush to lateral femoral cortex (binary Y/N)
- 8 cortex hold for plate screws (binary Y/N)

Sub- study 2: Hemiarthroplasty

1. Primary Outcome
    - Leg length discrepancy (mm)
2. Secondary Outcomes (in order of importance)
    - Femoral stem alignment (degrees off neutral)
    - Cement mantle quality (Barrack grade score)
    - Femoral offset change relative to native hip (mm)

Sub- study 3: Ankle fracture fixation

1. Primary Outcome
    - Medial clear space (mm)
2. Secondary Outcomes
    - Lateral malleolar displacement (mm)
    - Tibiofibular clear space (mm)
    - Talocrural angle (degrees)
    - Medial malleolar displacement (mm)

B) Clinical Outcomes

For peer review only

The clinical outcome measures for sub-studies 1-3 are;

1) Procedure Time

Defined as knife-to-skin/surgical start time to wound closure/surgical stop time. These will be obtained from hospital theatre management systems. Procedure time has been chosen as an outcome measure as there is evidence in the literature that procedure time is inversely related to experience, and so can be used as a surrogate measure of technical proficiency(14)

2) Intra-operative radiation dose to patient

Defined as time under fluoroscopy (seconds) and radiation dose (mGym2). There is evidence that with increasing experience and skill, surgeons use less intra-operative x-rays to adjust the position of the fracture and implant(14). Hemiarthroplasty does not require fluoroscopy so this will not be used as an outcome measure for sub-study 2.

3) Post-operative complication rate

The complications of interest are the acute post-operative complications during the inpatient admission. These will be sub-categorised as acute medical complications (hospital acquired pneumonia, renal complications, cardiac complications, DVT/PE) and surgical complications (wound infection, wound dehiscence, metalwork failure, deep infection).

For peer review only

4) Health state at 4 months post-operation (EQ-5D)

Health state at 4-months post-operation will be measured using EQ-5D, which is a standardized instrument measuring generic health status, which has been widely validated in clinical trials. This data is being collected separately as part of the WHiTE comprehensive cohort study of hip fracture patients (ISRCTN63982700) and reported elsewhere(15). EQ-5D will be used for sub-studies 1 and 2 only as these involve hip fractures.

Screening and eligibility

Orthopaedic surgeons-in-training in their first, second or third specialist training year in the West Midlands Workforce Deanery will be eligible to participate in the trial. Eligible trainees will be identified by liaison with the training programme directors for Trauma & Orthopaedic surgery in the three West Midlands schools; Warwick, Birmingham and Oswestry. An invitation email will be sent to all eligible trainees by programme administrators at the deanery.

Inclusion criteria

1) Trauma & Orthopaedic surgeon-in-training in West Midlands school (Warwick/Birmingham/Oswestry)

2) In specialty training year 1-3
3) Willing and able to attend a two-day cadaveric simulation training course at the West Midlands Surgical Training Centre, Coventry

Exclusion criteria

1) Unavailable on course dates

Consent

Surgeon participants:

Potential study participants will be provided with written and verbal information about the study. Consent will be obtained by the trial team. The right to refuse participation without giving reasons will be fully respected, and enrolled participants will be free to withdraw from the study at any time without reason, and without prejudice to their training. All participants will be provided with the contact information of a team member who can provide further information about the study. All participants who are allocated to the control group will have the opportunity to undertake the cadaveric simulation training intervention at the end of the study follow-up. This provision is being offered so that the control group are not disadvantaged in their access to educational opportunity by virtue of being randomised to the control group.

For peer review only

Patients whose operations are assessed:

Patients who undergo an operation by a surgeon who is participating in the study will not be separately consented to allow access to radiographs to assess their implant position or clinical outcome data. Permission to access this information for the purposes of this study without patient consent has been granted from the confidentiality advisory group (16/CAG/0125). It is recognised that seeking consent from a group of primarily elderly, frail patients to assess low risk, routine clinical data in a secure manner for a trial they are not directly participating in would be unduly burdensome for the patients. All patient data will be fully anonymised and handled securely in line with university data regulations.

Randomisation

Participants will be randomised at the point of recruitment using block randomisation (block size 4) to generate a random sequence list, to which participants will be assigned in the order that they enter the study. The allocation sequence will be generated by a senior medical statistician, participants will be enrolled by the trial team.

Postrandomisation withdrawals

Withdrawn participants will not be replaced.

Study setting

For peer review only

The study participants will be on training rotations within the regional hospitals of the West Midlands during the study follow-up. The hospitals where trainees have been working, and performing operations, during the study follow up will be identified from the participants electronic surgical logbook records.

Interventions

Control group

The control group will undertake standard residency training according to the master-apprentice model, which is the current standard practice in UK. No additional training or access to learning materials will be provided beyond the fortnightly didactic teaching sessions which are delivered as a part of routine training.

Intervention group (cadaveric simulation trained)

Participants allocated to the intervention group will receive an intensive, 2-day cadaveric simulation training course at the start of the training year, where 4 common orthopaedic surgical procedures will be taught (DHS, hemiarthroplasty, ankle fracture fixation and lower limb fasciotomy). All intervention participants will receive training on all 4 procedures, which will be considered separately in the analysis as individual sub-studies (as they have different radiographic outcome measures). The fasciotomy procedure is included as a 'filler' to make

For peer review only

the course structure work, and chosen because it is an important high-stakes, anatomically critical operation that is rarely performed by trainees. Outcomes related to the fasciotomy procedure will not be collected or included in the analysis.

The cadaveric simulation training course

The course will be delivered in September at the start of the surgical training year (which runs August to August). The course will take place at the West Midlands Surgical Training Centre (WMSTC) at the University Hospital Coventry & Warwickshire (UHCW). The WMSTC is a specialized wet-laboratory facility for delivering cadaveric training, and has an experienced dedicated faculty to facilitate training delivery.

The course will consist of two full days of teaching, with expert consultant faculty teaching on fresh-frozen hemi-cadavers (waist-to-toe-tip). The participant:faculty ratio will be 2:1, and participant:cadaver ratio will be 2:1. Each participant will undertake each of the four procedures in their entirety as primary surgeon('skin-to-skin'), and will act as assistant when their partner is the primary surgeon four times. Hence each participant is exposed to eight procedures during the course:

The environment and psychological fidelity of the simulation will be maximised by providing;

- Full surgical dress including masks, gloves, gowns and lead x-ray aprons

For peer review only

- The usual disposable surgical drapes
- Skin preparation (iodine solution) to prepare the surgical site, and participants and faculty will be asked to observe the usual sterile field precautions as in real theatre
- Full surgical instrument trays, surgical implants and cement (for hemiarthroplasty) of the same type as in real theatre will be used
- Image intensifier (mobile x-ray) will be available for intra-operative use
- Background noise levels and room temperature were maintained at what would usually be expected in the operating theatre

The simulated operating theatres will be set up within the WMSTC as two parallel round-robin circuits. The two stations requiring x-ray use (DHS and ankle ORIF) will be set up at the far end of the room to create a radiation zone and where appropriate, standard precautions will be used. Careful consideration will be given to the optimum sequential use of the cadaveric specimens in planning the course structure. For example, it is necessary that the DHS station precedes the hemiarthroplasty station as it would obviously not be possible to perform a DHS operation when the femoral head had been removed. Similarly, the fasciotomy incisions would compromise the soft tissue envelope of the lower limb to a sufficient degree that the fidelity of the ankle ORIF station would be compromised. It is important to make the best and most efficient use of the cadaveric material, for both ethical and financial reasons.

Blinding

For peer review only

The participants cannot be blinded to the type of training they receive, neither can the trial team in organising the cadaveric simulation training. The trial team will take no part in the training of participants. The assessment of radiographic images will be made blinded to group allocation.

Adverse event management

In the unlikely event of a serious adverse event (SAE), the chief investigator will report to the sponsor (University of Warwick), ethics committee and project supervisors.

Patient and public involvement

There was no direct patient or public involvement in the design of the study, although clearly training competent surgeons is in the public interest. There is qualitative work to be done around this trial to better understand patient expectations of surgical training.

End of trial

The trial will end when all the radiographic and clinical outcome data has been collected from the participating sites. The trial will be stopped prematurely if required by the ethics committee, following recommendations from the sponsor, or if funding for the study is withdrawn. The research ethics committee and confidentiality advisory group will be notified in writing once the trial is complete.

For peer review only

Trial Oversight

This trial is being undertaken as part of a doctoral research project (HKJ), and supervised by three senior supervisors (DG, JF, GTRP). The supervisors will act as the trial management group and steering committee. The trial is being conducted within a registered Clinical Trials Unit, and will follow the CTU standard operating procedures.

Data Collection Plan

Data on the numbers of procedures performed by the participating surgeons at baseline will be collected. The operations performed by the participants during study follow-up will be identified by the surgeons' electronic logbook. Only procedures coded as 'S-TS: supervised-trainer scrubbed' or 'STU: supervised trainer unscrubbed' will be included in the analysis. This is to ensure that only procedures where the trainee has performed the key parts (S-TS) or the entire procedure (STU) are included. If further information on supervisor input/takeover is required this can be obtained by accessing the corresponding procedure based assessment (PBA) record for the operation. PBAs are routinely collected as part of training.

Procedure data will be extracted and anonymised to study identifier by the electronic logbook data team, before being sent to the trial team. The data will include operation type, date, hospital, hospital ID, patient age, American Society of Anaesthesiologists Grade and supervision code. The radiographs and clinical outcome data relating to these procedures will then be obtained from the study sites via liaison with the respective Research &

For peer review only

Development Departments. Data will be entered into a secure trial database on a professionally encrypted trial-specific computer, fully anonymised with only study identifiers. Once data collection is complete, and prior to analysis, range checks for data values will be undertaken, and data will be double checked on entry to the statistical software package. The project supervisors will act as the data monitoring committee. No interim analysis will be undertaken. The trial team and statistician will have access to the final trial dataset.

For peer review only

Statistical Analysis Plan

Baseline data including completed months of training and number of prior procedures performed will be summarised and compared between the two arms of the study. A CONSORT chart showing the flow of participants through the study will be produced. The three taught procedures (substudies 1-3) will be analysed and reported individually.

The main analysis will investigate and report differences between the two groups with respect to the implant positions (as measured from radiographs), the procedure times, the intra-operative radiation dose to the patient, and patient outcomes, as measured by post-operative complications and health state at 4 months post-operation (hip fractures only).

Statistical tests will be two-sided and considered to demonstrate a significant difference when $p<0.05$. Temporal trends by group for implant position, procedure time and radiation dose will be presented. Linear mixed effects models will be fitted to allow for within-surgeon correlation between repeated observations (surgeon clustering as a random effect), and to adjust for important co-variates such as patient condition, age and surgeon experience.

These will be summarised by plotting individual learning curves, and then modelled to estimate the overall learning curves for the two arms of the study.

Descriptive statistical analyses of between-group comparisons will be presented for complication rate and health state, with temporal analysis of the latter being reported if appropriate and feasible. The statistical analysis will be supervised and checked by a senior medical statistician at Warwick University.

In the event of missing data, statistician advice will be sought on multiple imputation.

Ethics and dissemination

Master-apprentice ‘on-the-job’ training for surgeons is the current training standard in the UK(10, 16), and therefore the control arm of the study reflects usual practice. The cadaveric simulation training intervention is an experimental educational intervention and does not expose trial participants to any substantial risks of harm. The trial results will be reported in accordance with the CONSORT statement, and disseminated through publication in peer reviewed journals and conferences. The results of the trial will be presented to Health Education England and the Royal Surgical College. The dataset, statistical code and technical appendices will be made available on request to the corresponding author.

Funding

This work was supported by Versus Arthritis grant number 20845

For peer review only

Sponsor Contact Information

Mrs Jane Prewitt, University of Warwick, Research & Impact Services, University House,

Kirby Corner Road, Coventry, CV4 8UW

wmssponsorship@warwick.ac.uk

Declaration/Conflict of Interests

None to declare

References:

1. Temple J. Time for Training. A Review of the impact of the European Working Time Directive on the quality of training. London: Medical Education England; 2010.

For peer review only

2. Maxwell AJ, Crocker M, Jones TL, Bhagawati D, Papadopoulos MC, Bell BA. Implementation of the European Working Time Directive in neurosurgery reduces continuity of care and training opportunities. Acta Neurochirurgica. 2010;152(7):1207-10.
3. Pickersgill T. The European working time directive for doctors in training. BMJ. 2001;323(7324):1266.
4. Marron C, Byrnes C, Kirk S. An EWTD-compliant shift rota decreases training opportunities. Bulletin of the Royal College of Surgeons of England. 2005;87(7):246-8.
5. Training So. Report from the UK Shape of Training Steering Group (UKSTSG). General Medical Council; 2017.
6. England RCoSo. Improving Surgical Training - Trainee Prospectus. London: The Royal College of Surgeons; 2018.
7. Curran I. A Framework for Technology Enhanced Learning. London: Department of Health; 2011.
8. James H, Chapman A, Pattison G, Griffin DR, Fisher J. Current status of Cadaveric Simulation for surgical training: a Systematic Review. British Journal of Surgery. 2019.
9. Gilbody J, Prasthofer A, Ho K, Costa M. The use and effectiveness of cadaveric workshops in higher surgical training: a systematic review. Annals of the Royal College of Surgeons of England. 2011;93(5):347-56.
10. Reznick RK, MacRae H. Teaching surgical skills--changes in the wind. N Engl J Med. 2006;355(25):2664-9.
11. James H, Pattison G, Griffin D, Fisher J. How does cadaveric simulation influence learning in Orthopedic residents? Journal of Surgical Education. 2020;In Press.
12. Schulz KF, Altman DG, Moher D. CONSORT 2010 Statement: updated guidelines for reporting parallel group randomised trials. BMJ. 2010;340.
13. Chan A-W, Tetzlaff JM, Altman DG, Laupacis A, Gøtzsche PC, Krleža-Jerić K, et al. SPIRIT 2013 Statement: Defining Standard Protocol Items for Clinical Trials. Annals of Internal Medicine. 2013;158(3):200-7.
14. James H, Chapman A, Pattison G, Fisher JG, Griffin DR. Assessment of technical skill acquisition and operative competence in Trauma and Orthopaedic surgical training: A systematic review. JBJS(Am) Reviews. 2019.
15. Costa ML, Griffin XL, Achten J, Metcalfe D, Judge A, Pinedo-Villanueva R, et al. World Hip Trauma Evaluation (WHiTE): framework for embedded comprehensive cohort studies. BMJ Open. 2016;6(10):e011679.
16. Hargreaves DH, Bowditch MG, Griffin DR. On-the-job training for surgeons. 1 ed. London: The Royal Society of Medicine Press; 1997.

Author Statement/Contributions

HKJ designed the study and wrote the manuscript

GTRP co-designed the study and the intervention and edited the manuscript

JDF edited the manuscript, made a substantial contribution to the design and is lead supervisor for the qualitative part of the project

For peer review only

DG co-designed the study, edited draft protocols and is lead supervisor for the quantitative part of the project

Acknowledgements

None

For peer review only

cad:trauma study

THE UNIVERSITY OF WARWICK

CONSENT FORM

(Biomedical and Scientific Research Ethics Committee) Study Number: REGO-2014-718

Title of Project: cad:trauma study

Name of Researcher(s): Mrs Hannah James, Professor Damian Griffin, Mr Giles Pattison, Dr Jane Kidd

Please initial all boxes

1. I confirm that I have read and understand the information sheet dated **April 2014** (version **1)** for the above study. I have had the opportunity to consider the information, ask questions and have had these answered satisfactorily. ☐
2. I understand that my participation is voluntary and that I am free to withdraw at any time without giving any reason, without my education or legal rights being affected. ☐
3. I understand that relevant sections of data collected during the study,may be looked at by individuals from The University of Warwick or from regulatory authorities where it is relevant. I give permission for these individuals to have access to this data. ☐
4. I agree to take part in the above study. ☐

Name of Participant	Date	Signature
Name of Person taking consent	Date	Signature

BSREC Consent form template; version number: 2012-13.01; Version date: 01Oct12
Investigator's consent form date of issue: [DATE]
Investigator's consent form version number: [VERSION]

For peer review only - http://bmjopen.bmj.com/site/about/guidelines.xhtml

Reporting checklist for protocol of a clinical trial.

Based on the SPIRIT guidelines.

Instructions to authors

Complete this checklist by entering the page numbers from your manuscript where readers will find each of the items listed below.

Your article may not currently address all the items on the checklist. Please modify your text to include the missing information. If you are certain that an item does not apply, please write "n/a" and provide a short explanation.

Upload your completed checklist as an extra file when you submit to a journal.

In your methods section, say that you used the SPIRITreporting guidelines, and cite them as:

Chan A-W, Tetzlaff JM, Altman DG, Laupacis A, Gøtzsche PC, Krleža-Jerić K, Hróbjartsson A, Mann H, Dickersin K, Berlin J, Doré C, Parulekar W, Summerskill W, Groves T, Schulz K, Sox H, Rockhold FW, Rennie D, Moher D. SPIRIT 2013 Statement: Defining standard protocol items for clinical trials. Ann Intern Med. 2013;158(3):200-207

		Reporting Item	Page Number
**Administrative information**			
Title	#1	Descriptive title identifying the study design, population, interventions, and, if applicable, trial acronym	1
Trial registration	#2a	Trial identifier and registry name. If not yet registered, name of intended registry	3
Trial registration: data set	#2b	All items from the World Health Organization Trial Registration Data Set	3
Protocol version	#3	Date and version identifier	3
Funding	#4	Sources and types of financial, material, and other support	15
Roles and responsibilities: contributorship	#5a	Names, affiliations, and roles of protocol contributors	1

For peer review only

Roles and responsibilities: sponsor contact information	#5b	Name and contact information for the trial sponsor	15
Roles and responsibilities: sponsor and funder	#5c	Role of study sponsor and funders, if any, in study design; collection, management, analysis, and interpretation of data; writing of the report; and the decision to submit the report for publication, including whether they will have ultimate authority over any of these activities	14
Roles and responsibilities: committees	#5d	Composition, roles, and responsibilities of the coordinating centre, steering committee, endpoint adjudication committee, data management team, and other individuals or groups overseeing the trial, if applicable (see Item 21a for data monitoring committee)	14
**Introduction**			
Background and rationale	#6a	Description of research question and justification for undertaking the trial, including summary of relevant studies (published and unpublished) examining benefits and harms for each intervention	1
Background and rationale: choice of comparators	#6b	Explanation for choice of comparators	11
Objectives	#7	Specific objectives or hypotheses	5-6
Trial design	#8	Description of trial design including type of trial (eg, parallel group, crossover, factorial, single group), allocation ratio, and framework (eg, superiority, equivalence, non-inferiority, exploratory)	5
**Methods: Participants, interventions, and outcomes**			
Study setting	#9	Description of study settings (eg, community clinic, academic hospital) and list of countries where data will be collected. Reference to where list of study sites can be obtained	10

For peer review only

Eligibility criteria	#10	Inclusion and exclusion criteria for participants. If applicable, eligibility criteria for study centres and individuals who will perform the interventions (eg, surgeons, psychotherapists)	9
Interventions: description	#11a	Interventions for each group with sufficient detail to allow replication, including how and when they will be administered	11-13
Interventions: modifications	#11b	Criteria for discontinuing or modifying allocated interventions for a given trial participant (eg, drug dose change in response to harms, participant request, or improving / worsening disease)	N/A – educational trial
Interventions: adherance	#11c	Strategies to improve adherence to intervention protocols, and any procedures for monitoring adherence (eg, drug tablet return; laboratory tests)	N/A – educational trial
Interventions: concomitant care	#11d	Relevant concomitant care and interventions that are permitted or prohibited during the trial	N/A – educational trial
Outcomes	#12	Primary, secondary, and other outcomes, including the specific measurement variable (eg, systolic blood pressure), analysis metric (eg, change from baseline, final value, time to event), method of aggregation (eg, median, proportion), and time point for each outcome. Explanation of the clinical relevance of chosen efficacy and harm outcomes is strongly recommended	6-7
Participant timeline	#13	Time schedule of enrolment, interventions (including any run-ins and washouts), assessments, and visits for participants. A schematic diagram is highly recommended (see Figure)	11
Sample size	#14	Estimated number of participants needed to achieve study objectives and how it was determined, including clinical and statistical assumptions supporting any sample size calculations	6
Recruitment	#15	Strategies for achieving adequate participant enrolment to reach target sample size	8

For peer review only

**Methods: Assignment of interventions (for controlled trials)**			
Allocation: sequence generation	#16a	Method of generating the allocation sequence (eg, computer-generated random numbers), and list of any factors for stratification. To reduce predictability of a random sequence, details of any planned restriction (eg, blocking) should be provided in a separate document that is unavailable to those who enrol participants or assign interventions	10
Allocation concealment mechanism	#16b	Mechanism of implementing the allocation sequence (eg, central telephone; sequentially numbered, opaque, sealed envelopes), describing any steps to conceal the sequence until interventions are assigned	10
Allocation: implementation	#16c	Who will generate the allocation sequence, who will enrol participants, and who will assign participants to interventions	10
Blinding (masking)	#17a	Who will be blinded after assignment to interventions (eg, trial participants, care providers, outcome assessors, data analysts), and how	13
Blinding (masking): emergency unblinding	#17b	If blinded, circumstances under which unblinding is permissible, and procedure for revealing a participant's allocated intervention during the trial	N/A - educational trial
**Methods: Data collection, management, and analysis**			
Data collection plan	#18a	Plans for assessment and collection of outcome, baseline, and other trial data, including any related processes to promote data quality (eg, duplicate measurements, training of assessors) and a description of study instruments (eg, questionnaires, laboratory tests) along with their reliability and validity, if known. Reference to where data collection forms can be found, if not in the protocol	14
Data collection plan: retention	#18b	Plans to promote participant retention and complete follow-up, including list of any outcome data to be collected for	14

For peer review only

		participants who discontinue or deviate from intervention protocols	
Data management	#19	Plans for data entry, coding, security, and storage, including any related processes to promote data quality (eg, double data entry; range checks for data values). Reference to where details of data management procedures can be found, if not in the protocol	14
Statistics: outcomes	#20a	Statistical methods for analysing primary and secondary outcomes. Reference to where other details of the statistical analysis plan can be found, if not in the protocol	14-15
Statistics: additional analyses	#20b	Methods for any additional analyses (eg, subgroup and adjusted analyses)	14-15
Statistics: analysis population and missing data	#20c	Definition of analysis population relating to protocol non-adherence (eg, as randomised analysis), and any statistical methods to handle missing data (eg, multiple imputation)	15
**Methods: Monitoring**			
Data monitoring: formal committee	#21a	Composition of data monitoring committee (DMC); summary of its role and reporting structure; statement of whether it is independent from the sponsor and competing interests; and reference to where further details about its charter can be found, if not in the protocol. Alternatively, an explanation of why a DMC is not needed	14
Data monitoring: interim analysis	#21b	Description of any interim analyses and stopping guidelines, including who will have access to these interim results and make the final decision to terminate the trial	14
Harms	#22	Plans for collecting, assessing, reporting, and managing solicited and spontaneously reported adverse events and other unintended effects of trial interventions or trial conduct	N/A – educational trial
Auditing	#23	Frequency and procedures for auditing trial conduct, if any, and whether the process will be independent from investigators and the sponsor	N/A – educational trial
**Ethics and dissemination**			

For peer review only

Research ethics approval	#24	Plans for seeking research ethics committee / institutional review board (REC / IRB) approval	3
Protocol amendments	#25	Plans for communicating important protocol modifications (eg, changes to eligibility criteria, outcomes, analyses) to relevant parties (eg, investigators, REC / IRBs, trial participants, trial registries, journals, regulators)	N/A – educational trial
Consent or assent	#26a	Who will obtain informed consent or assent from potential trial participants or authorised surrogates, and how (see Item 32)	9
Consent or assent: ancillary studies	#26b	Additional consent provisions for collection and use of participant data and biological specimens in ancillary studies, if applicable	N/A – educational trial
Confidentiality	#27	How personal information about potential and enrolled participants will be collected, shared, and maintained in order to protect confidentiality before, during, and after the trial	14
Declaration of interests	#28	Financial and other competing interests for principal investigators for the overall trial and each study site	16
Data access	#29	Statement of who will have access to the final trial dataset, and disclosure of contractual agreements that limit such access for investigators	14
Ancillary and post trial care	#30	Provisions, if any, for ancillary and post-trial care, and for compensation to those who suffer harm from trial participation	N/A – educational trial
Dissemination policy: trial results	#31a	Plans for investigators and sponsor to communicate trial results to participants, healthcare professionals, the public, and other relevant groups (eg, via publication, reporting in results databases, or other data sharing arrangements), including any publication restrictions	15
Dissemination policy: authorship	#31b	Authorship eligibility guidelines and any intended use of professional writers	15
Dissemination policy: reproducible research	#31c	Plans, if any, for granting public access to the full protocol, participant-level dataset, and statistical code	N/A

Appendices

For peer review only

Informed consent materials	#32	Model consent form and other related documentation given to participants and authorised surrogates	Appendix
Biological specimens	#33	Plans for collection, laboratory evaluation, and storage of biological specimens for genetic or molecular analysis in the current trial and for future use in ancillary studies, if applicable	N/A – educational trial

None The SPIRIT checklist is distributed under the terms of the Creative Commons Attribution License CC-BY-ND 3.0. This checklist can be completed online using https://www.goodreports.org/, a tool made by the EQUATOR Network in collaboration with Penelope.ai

For peer review only

BMJ Open

Cadaveric simulation vs standard training for postgraduate trauma & orthopaedic surgical trainees: protocol for the CAD:TRAUMA study multi-centre randomised controlled educational trial

Journal:	*BMJ Open*
Manuscript ID	bmjopen-2020-037319.R2
Article Type:	Protocol
Date Submitted by the Author:	22-Jul-2020
Complete List of Authors:	James, Hannah; University of Warwick Warwick Medical School, Clinical Trials Unit; University Hospitals Coventry and Warwickshire NHS Trust, Trauma & Orthopaedic Surgery<br>Pattison, Giles; University Hospitals Coventry and Warwickshire NHS Trust, Trauma & Orthopaedic Surgery<br>Fisher, Joanne; University of Warwick Warwick Medical School, Clinical Trials Unit<br>Griffin, Damian; University of Warwick, Warwick Medical School
**Primary Subject Heading**:	Medical education and training
Secondary Subject Heading:	Surgery
Keywords:	MEDICAL EDUCATION & TRAINING, ORTHOPAEDIC & TRAUMA SURGERY, Trauma management < ORTHOPAEDIC & TRAUMA SURGERY

SCHOLARONE™
Manuscripts

I, the Submitting Author has the right to grant and does grant on behalf of all authors of the Work (as defined in the below author licence), an exclusive licence and/or a non-exclusive licence for contributions from authors who are: i) UK Crown employees; ii) where BMJ has agreed a CC-BY licence shall apply, and/or iii) in accordance with the terms applicable for US Federal Government officers or employees acting as part of their official duties; on a worldwide, perpetual, irrevocable, royalty-free basis to BMJ Publishing Group Ltd ("BMJ") its licensees and where the relevant Journal is co-owned by BMJ to the co-owners of the Journal, to publish the Work in this journal and any other BMJ products and to exploit all rights, as set out in our licence.

The Submitting Author accepts and understands that any supply made under these terms is made by BMJ to the Submitting Author unless you are acting as an employee on behalf of your employer or a postgraduate student of an affiliated institution which is paying any applicable article publishing charge ("APC") for Open Access articles. Where the Submitting Author wishes to make the Work available on an Open Access basis (and intends to pay the relevant APC), the terms of reuse of such Open Access shall be governed by a Creative Commons licence – details of these licences and which Creative Commons licence will apply to this Work are set out in our licence referred to above.

Other than as permitted in any relevant BMJ Author's Self Archiving Policies, I confirm this Work has not been accepted for publication elsewhere, is not being considered for publication elsewhere and does not duplicate material already published. I confirm all authors consent to publication of this Work and authorise the granting of this licence.

For peer review only

Cadaveric simulation vs standard training for postgraduate trauma & orthopaedic surgical trainees: protocol for the CAD:TRAUMA study multi-centre randomised controlled educational trial

Hannah K James[1,2] h.smith.1@warwick.ac.uk

Giles T R Pattison[2] Giles.Pattison@uhcw.nhs.uk

Joanne D Fisher[1] Joanne.Fisher@warwick.ac.uk

Damian R Griffin[1,2] Damian.Griffin@warwick.ac.uk

1. Clinical Trials Unit, Warwick Medical School, Coventry, CV4 7HL
2. Department of Trauma & Orthopaedic Surgery, University Hospitals Coventry & Warwickshire, Clifford Bridge Road, Coventry, CV2 2DX

Correspondence:

Dr HK James

For peer review only

Clinical Sciences Research Laboratories

Clinical Sciences Building

University Hospital Coventry & Warwickshire

Clifford Bridge Road

Coventry

CV2 2DX

Tel 07786737479

Word Count 3,420

Keywords

Education, Training, Simulation

For peer review only

Abstract

Introduction

The quantity and quality of surgical training in the UK has been negatively affected by reduced working hours and NHS financial pressures. Traditionally surgical training has occurred by the master-apprentice model involving a process of graduated responsibility, but a modern alternative is to use simulation for the early stages of training. It is not known if simulation training for junior trainees can safeguard patients and improve clinical outcomes.

This paper details the protocol for a multicentre randomised controlled educational trial of a cadaveric simulation training intervention versus standard training for junior postgraduate orthopaedic surgeons-in-training. This is the first study to assess the effect of cadaveric simulation training for open surgery on patient outcome. The feasibility of delivering cadaveric training, use of radiographic and clinical outcome measures to assess impact and the challenges of upscaling provision will be explored.

Methods and Analysis

We will recruit postgraduate orthopaedic surgeons-in-training in the first three years (of eight) of the specialist training programme. Participants will be block randomised and allocated to either cadaveric simulation or standard 'on-the-job' training, learning three common orthopaedic procedures, each of which is a sub-study within the trial. The procedures are 1) Dynamic Hip Screw(DHS), 2) hemiarthroplasty and 3) ankle fracture fixation. These procedures have been selected as they are very common procedures which are routinely performed by junior surgeons-in-training. A pragmatic approach to sample size is taken in lieu of a formal power calculation as this is novel exploratory work with no a priori estimate of effect size to reference. The primary outcome measure is the technical success of the surgery performed on patients by the participating surgeons-in-training during the follow-up period for the three sub-study procedures, as measured by the implant position on the post-operative radiograph. The secondary outcome measures are procedure time, post-operative complication rate and patient health state at 4 months post-operation (EQ-5D – substudies 1 and 2 only).

For peer review only

Ethics, registration and dissemination

National research ethics approval was granted for this study by the NHS Research Authority South Birmingham Research Ethics Committee (15/WM/0464). Confidentiality Advisory Group approval was granted for accessing radiographic and outcome data without patient consent on 27 February 2017 (16/CAG/0125). The results of this trial will be submitted to a peer-reviewed journal and will inform educational and clinical practice.

Trial registration number

ISRCTN20431944; Pre-results

Protocol version V_P_1.1

Article Summary

Strengths and limitations of this study:

- This is the first randomised controlled trial assessing the impact of cadaveric simulation training on clinical outcomes
- Patient-centred outcome measures are used to measure an educational intervention for surgeons

For peer review only

- Multicentre study to maximize external validity of the results
- The training dose is small as cadaveric training is expense to deliver
- Pragmatic approach to sample size which is limited by the capacity of the surgical training centre

For peer review only

Introduction

It is imperative that surgeons are trained to a high standard, so they can perform safe and effective operations for patients. The quality and quantity of surgical training in the UK is currently under threat from a ‘perfect storm’ of factors(1). These include reduced working hours(2, 3), shift based working patterns(4) with the loss of the traditional surgical firm, and a move to expedite training and shorten specialist programmes(5, 6). This is set within a climate of unprecedented financial austerity in the NHS and ever increasing service pressures.

Simulation offers a solution to some of these challenges by moving the early part of the surgical learning curve away from patients into a controlled environment(7), where skills may be more rapidly acquired as compared to the clinical environment. Simulation is also

potentially a very efficient way of training, as large numbers of trainees can be trained simultaneously, at an intensity not feasible in the clinical environment due to competing service demands.

Cadaveric simulation - training using deceased, preserved or fresh human bodies - is a particularly promising modality for training. Fresh-frozen cadavers retain many of the soft tissue handling characteristics seen in live patients, and in combination with presenting the correct anatomy, particularly complex neurovascular relationships, may offer a more realistic simulated operation than would be possible on a plastic model or virtual reality simulator(8, 9). Cadaveric material does not bleed(10) and hence may be less useful for simulating procedures where haemorrhage control is an important feature.

The operating theatre environment can be simulated, including (but not limited to) surgical dress, draping, instrumentation and multidisciplinary team. This 'whole dress rehearsal' for surgery may enhance development of non-technical skills in addition to the technical operative surgical skills(11).

There are several challenges in delivering cadaveric simulation training. It is expensive to provide(9), particularly when cadaveric material has to be purchased under license where there is not a local body donation programme. It requires considerable infrastructure to deliver, including specialist wet laboratory facilities with the appropriately trained staff. These challenges become particularly pressing when provision of cadaveric training on a large scale is considered, and are an important driver in the development of high quality evidence

For peer review only

of educational impact. This evidence is necessary before considerable financial investment can be recommended in providing cadaveric simulation training on a larger scale.

There is abundant low quality evidence showing cadaveric simulation may induce short term skill improvement as measured by subjective and behavioural metrics, but there is a lack of high quality, quantitative evidence that skills learnt in cadaveric simulation can transfer to the workplace, leading to improved outcomes for patients(8).

Our trial attempts to address this evidence deficit, and is both topical and timely.

<u>Good Clinical Practice</u>

This trial will be undertaken in compliance with Good Practice Guidelines, complying with the Declaration of Helsinki and UK Legislation. Warwick standard operating procedures (SOPs) will be followed.

<u>Consolidated Standards of Reporting Trials</u>

The results of the trial will be reported in line with the Consolidated Standard of Reporting (CONSORT) statement(12). This protocol has been written according to the SPIRIT reporting guidelines(13).

<u>Aim</u>

For peer review only

The aim is to determine which of the two surgical training strategies for junior orthopaedic surgeons-in-training leads to the best patient outcomes for three common procedures.

Objectives

1. To assess the impact of a cadaveric simulation training intervention on the patient outcome of operations performed by junior orthopaedic surgeons-in-training
2. To define the early learning curve of DHS, Hemiarthroplasty and ankle fracture fixation
3. To explore the feasibility of using post-operative x-rays to assess technical skill

Methods and Analysis

Study design

This is a UK multicentre, two-arm, group parallel randomised controlled educational trial

Sample size

For peer review only

This trial is the first attempt to objectively measure transfer of open operative skills from cadaveric simulation into the workplace using patient-based outcome measures. There is no available estimate of effect size to reference against a priori in determining sample size, therefore a pragmatic approach to sample size will be taken in lieu of a formal power calculation. The surgical training centre can accommodate 16 delegates at one time and financial resources permitted one iteration of the cadaveric training course. Our maximum sample size is therefore 16 participants in each arm of the study

Outcome measures

A) Radiographic outcomes

The radiographs will be obtained electronically from hospital servers and the implant position measured manually using computer software. The operations will be identified retrospectively by access to the participating surgeons' electronic logbooks. The measurements vary by operation type and are defined as follows.

Sub-study 1: Dynamic Hip Screw

1. Primary Outcome
   - Tip-Apex distance (in mm)
2. Secondary Outcomes (in order of importance)
   - Lag screw position in the femoral head (defined by Cleveland Zones)

For peer review only

- Plate flush to lateral femoral cortex (binary Y/N)
- 8 cortex hold for plate screws (binary Y/N)

Sub- study 2: Hemiarthroplasty

1. Primary Outcome
    - Leg length discrepancy (mm)
2. Secondary Outcomes (in order of importance)
    - Femoral stem alignment (degrees off neutral)
    - Cement mantle quality (Barrack grade score)
    - Femoral offset change relative to native hip (mm)

Sub- study 3: Ankle fracture fixation

1. Primary Outcome
    - Medial clear space (mm)
2. Secondary Outcomes
    - Lateral malleolar displacement (mm)
    - Tibiofibular clear space (mm)
    - Talocrural angle (degrees)
    - Medial malleolar displacement (mm)

B) Clinical Outcomes

For peer review only

The clinical outcome measures for sub-studies 1-3 are;

1) Procedure Time

Defined as knife-to-skin/surgical start time to wound closure/surgical stop time. These will be obtained from hospital theatre management systems. Procedure time has been chosen as an outcome measure as there is evidence in the literature that procedure time is inversely related to experience, and so can be used as a surrogate measure of technical proficiency(14)

2) Intra-operative radiation dose to patient

Defined as time under fluoroscopy (seconds) and radiation dose (mGym2). There is evidence that with increasing experience and skill, surgeons use less intra-operative x-rays to adjust the position of the fracture and implant(14). Hemiarthroplasty does not require fluoroscopy so this will not be used as an outcome measure for sub-study 2.

3) Post-operative complication rate

The complications of interest are the acute post-operative complications during the inpatient admission. These will be sub-categorised as acute medical complications (hospital acquired pneumonia, renal complications, cardiac complications, DVT/PE) and surgical complications (wound infection, wound dehiscence, metalwork failure, deep infection).

For peer review only

4) Health state at 4 months post-operation (EQ-5D)

Health state at 4-months post-operation will be measured using EQ-5D, which is a standardized instrument measuring generic health status, which has been widely validated in clinical trials. This data is being collected separately as part of the WHiTE comprehensive cohort study of hip fracture patients (ISRCTN63982700) and reported elsewhere(15). EQ-5D will be used for sub-studies 1 and 2 only as these involve hip fractures.

Screening and eligibility

Orthopaedic surgeons-in-training in their first, second or third specialist training year in the West Midlands Workforce Deanery will be eligible to participate in the trial. Eligible trainees will be identified by liaison with the training programme directors for Trauma & Orthopaedic surgery in the three West Midlands schools; Warwick, Birmingham and Oswestry. An invitation email will be sent to all eligible trainees by programme administrators at the deanery.

Inclusion criteria

1) Trauma & Orthopaedic surgeon-in-training in West Midlands school (Warwick/Birmingham/Oswestry)

For peer review only

2) In specialty training year 1-3
3) Willing and able to attend a two-day cadaveric simulation training course at the West Midlands Surgical Training Centre, Coventry

Exclusion criteria

1) Unavailable on course dates

Consent

Surgeon participants:

Potential study participants will be provided with written and verbal information about the study. Consent will be obtained by the trial team. The right to refuse participation without giving reasons will be fully respected, and enrolled participants will be free to withdraw from the study at any time without reason, and without prejudice to their training. All participants will be provided with the contact information of a team member who can provide further information about the study. All participants who are allocated to the control group will have the opportunity to undertake the cadaveric simulation training intervention at the end of the study follow-up. This provision is being offered so that the control group are not disadvantaged in their access to educational opportunity by virtue of being randomised to the control group.

For peer review only

Patients whose operations are assessed:

Patients who undergo an operation by a surgeon who is participating in the study will not be separately consented to allow access to radiographs to assess their implant position or clinical outcome data. Permission to access this information for the purposes of this study without patient consent has been granted from the confidentiality advisory group (16/CAG/0125). It is recognised that seeking consent from a group of primarily elderly, frail patients to assess low risk, routine clinical data in a secure manner for a trial they are not directly participating in would be unduly burdensome for the patients. All patient data will be fully anonymised and handled securely in line with university data regulations.

Randomisation

Participants will be randomised at the point of recruitment using block randomisation (block size 4) to generate a random sequence list, to which participants will be assigned in the order that they enter the study. The allocation sequence will be generated by a senior medical statistician, participants will be enrolled by the trial team.

Postrandomisation withdrawals

Withdrawn participants will not be replaced.

Study setting

For peer review only

The study participants will be on training rotations within the regional hospitals of the West Midlands during the study follow-up. The hospitals where trainees have been working, and performing operations, during the study follow up will be identified from the participants electronic surgical logbook records.

Interventions

Control group

The control group will undertake standard residency training according to the master-apprentice model, which is the current standard practice in UK. No additional training or access to learning materials will be provided beyond the fortnightly didactic teaching sessions which are delivered as a part of routine training.

Intervention group (cadaveric simulation trained)

Participants allocated to the intervention group will receive an intensive, 2-day cadaveric simulation training course at the start of the training year, where 4 common orthopaedic surgical procedures will be taught (DHS, hemiarthroplasty, ankle fracture fixation and lower limb fasciotomy). All intervention participants will receive training on all 4 procedures, which will be considered separately in the analysis as individual sub-studies (as they have different radiographic outcome measures). The fasciotomy procedure is included as a ‘filler’ to make

For peer review only

the course structure work, and chosen because it is an important high-stakes, anatomically critical operation that is rarely performed by trainees. Outcomes related to the fasciotomy procedure will not be collected or included in the analysis.

The cadaveric simulation training course

The course will be delivered in September at the start of the surgical training year (which runs August to August). The course will take place at the West Midlands Surgical Training Centre (WMSTC) at the University Hospital Coventry & Warwickshire (UHCW). The WMSTC is a specialized wet-laboratory facility for delivering cadaveric training, and has an experienced dedicated faculty to facilitate training delivery.

The course will consist of two full days of teaching, with expert consultant faculty teaching on fresh-frozen hemi-cadavers (waist-to-toe-tip). The participant:faculty ratio will be 2:1, and participant:cadaver ratio will be 2:1. Each participant will undertake each of the four procedures in their entirety as primary surgeon('skin-to-skin'), and will act as assistant when their partner is the primary surgeon four times. Hence each participant is exposed to eight procedures during the course:

The environment and psychological fidelity of the simulation will be maximised by providing;

- Full surgical dress including masks, gloves, gowns and lead x-ray aprons

For peer review only

- The usual disposable surgical drapes
- Skin preparation (iodine solution) to prepare the surgical site, and participants and faculty will be asked to observe the usual sterile field precautions as in real theatre
- Full surgical instrument trays, surgical implants and cement (for hemiarthroplasty) of the same type as in real theatre will be used
- Image intensifier (mobile x-ray) will be available for intra-operative use
- Background noise levels and room temperature were maintained at what would usually be expected in the operating theatre

The simulated operating theatres will be set up within the WMSTC as two parallel round-robin circuits. The two stations requiring x-ray use (DHS and ankle ORIF) will be set up at the far end of the room to create a radiation zone and where appropriate, standard precautions will be used. Careful consideration will be given to the optimum sequential use of the cadaveric specimens in planning the course structure. For example, it is necessary that the DHS station precedes the hemiarthroplasty station as it would obviously not be possible to perform a DHS operation when the femoral head had been removed. Similarly, the fasciotomy incisions would compromise the soft tissue envelope of the lower limb to a sufficient degree that the fidelity of the ankle ORIF station would be compromised. It is important to make the best and most efficient use of the cadaveric material, for both ethical and financial reasons.

Blinding

For peer review only

The participants cannot be blinded to the type of training they receive, neither can the trial team in organising the cadaveric simulation training. The trial team will take no part in the training of participants. The assessment of radiographic images will be made blinded to group allocation.

Adverse event management

In the unlikely event of a serious adverse event (SAE), the chief investigator will report to the sponsor (University of Warwick), ethics committee and project supervisors.

Patient and public involvement

There was no direct patient or public involvement in the design of the study, although clearly training competent surgeons is in the public interest. There is qualitative work to be done around this trial to better understand patient expectations of surgical training.

End of trial

The trial will end when all the radiographic and clinical outcome data has been collected from the participating sites. The trial will be stopped prematurely if required by the ethics committee, following recommendations from the sponsor, or if funding for the study is withdrawn. The research ethics committee and confidentiality advisory group will be notified in writing once the trial is complete.

For peer review only

Trial Oversight

This trial is being undertaken as part of a doctoral research project (HKJ), and supervised by three senior supervisors (DG, JF, GTRP). The supervisors will act as the trial management group and steering committee. The trial is being conducted within a registered Clinical Trials Unit, and will follow the CTU standard operating procedures.

For peer review only

Data Collection Plan

Data on the numbers of procedures performed by the participating surgeons at baseline will be collected. The operations performed by the participants during study follow-up will be identified by the surgeons' electronic logbook. Only procedures coded as 'S-TS: supervised-trainer scrubbed' or 'STU: supervised trainer unscrubbed' will be included in the analysis. This is to ensure that only procedures where the trainee has performed the key parts (S-TS) or the entire procedure (STU) are included. If further information on supervisor input/takeover is required this can be obtained by accessing the corresponding procedure based assessment (PBA) record for the operation. PBAs are routinely collected as part of training.

Procedure data will be extracted and anonymised to study identifier by the electronic logbook data team, before being sent to the trial team. The data will include operation type, date, hospital, hospital ID, patient age, American Society of Anaesthesiologists Grade and supervision code. The radiographs and clinical outcome data relating to these procedures will then be obtained from the study sites via liaison with the respective Research &

Development Departments. Data will be entered into a secure trial database on a professionally encrypted trial-specific computer, fully anonymised with only study identifiers. Once data collection is complete, and prior to analysis, range checks for data values will be undertaken, and data will be double checked on entry to the statistical software package. The project supervisors will act as the data monitoring committee. No interim analysis will be undertaken. The trial team and statistician will have access to the final trial dataset.

Statistical Analysis Plan

Baseline data including completed months of training and number of prior procedures performed will be summarised and compared between the two arms of the study. A CONSORT chart showing the flow of participants through the study will be produced. The three taught procedures (substudies 1-3) will be analysed and reported individually.

The main analysis will investigate and report differences between the two groups with respect to the implant positions (as measured from radiographs), the procedure times, the intra-operative radiation dose to the patient, and patient outcomes, as measured by post-operative complications and health state at 4 months post-operation (hip fractures only).

Statistical tests will be two-sided and considered to demonstrate a significant difference when $p<0.05$. Temporal trends by group for implant position, procedure time and radiation dose will be presented. Linear mixed effects models will be fitted to allow for within-surgeon correlation between repeated observations (surgeon clustering as a random effect), and to adjust for important co-variates such as patient condition, age and surgeon experience.

For peer review only

These will be summarised by plotting individual learning curves, and then modelled to estimate the overall learning curves for the two arms of the study.

Descriptive statistical analyses of between-group comparisons will be presented for complication rate and health state, with temporal analysis of the latter being reported if appropriate and feasible. The statistical analysis will be supervised and checked by a senior medical statistician at Warwick University.

In the event of missing data, statistician advice will be sought on multiple imputation.

Ethics and dissemination

Master-apprentice 'on-the-job' training for surgeons is the current training standard in the UK(10, 16), and therefore the control arm of the study reflects usual practice. The cadaveric simulation training intervention is an experimental educational intervention and does not expose trial participants to any substantial risks of harm. The trial results will be reported in accordance with the CONSORT statement, and disseminated through publication in peer reviewed journals and conferences. The results of the trial will be presented to Health Education England and the Royal Surgical College. The dataset, statistical code and technical appendices will be made available on request to the corresponding author. The study was approved by the NHS Research Authority South Birmingham Research Ethics Committee (15/WM/0464).

Funding

For peer review only

This work was supported by Versus Arthritis grant number 20845

Sponsor Contact Information

Mrs Jane Prewitt, University of Warwick, Research & Impact Services, University House, Kirby Corner Road, Coventry, CV4 8UW

wmssponsorship@warwick.ac.uk

Declaration/Conflict of Interests

None to declare

References:

For peer review only

1. Temple J. Time for Training. A Review of the impact of the European Working Time Directive on the quality of training. London: Medical Education England; 2010.
2. Maxwell AJ, Crocker M, Jones TL, Bhagawati D, Papadopoulos MC, Bell BA. Implementation of the European Working Time Directive in neurosurgery reduces continuity of care and training opportunities. Acta Neurochirurgica. 2010;152(7):1207-10.
3. Pickersgill T. The European working time directive for doctors in training. BMJ. 2001;323(7324):1266.
4. Marron C, Byrnes C, Kirk S. An EWTD-compliant shift rota decreases training opportunities. Bulletin of the Royal College of Surgeons of England. 2005;87(7):246-8.
5. Training So. Report from the UK Shape of Training Steering Group (UKSTSG). General Medical Council; 2017.
6. England RCoSo. Improving Surgical Training - Trainee Prospectus. London: The Royal College of Surgeons; 2018.
7. Curran I. A Framework for Technology Enhanced Learning. London: Department of Health; 2011.
8. James H, Chapman A, Pattison G, Griffin DR, Fisher J. Current status of Cadaveric Simulation for surgical training: a Systematic Review. British Journal of Surgery. 2019.
9. Gilbody J, Prasthofer A, Ho K, Costa M. The use and effectiveness of cadaveric workshops in higher surgical training: a systematic review. Annals of the Royal College of Surgeons of England. 2011;93(5):347-56.
10. Reznick RK, MacRae H. Teaching surgical skills--changes in the wind. N Engl J Med. 2006;355(25):2664-9.
11. James H, Pattison G, Griffin D, Fisher J. How does cadaveric simulation influence learning in Orthopedic residents? Journal of Surgical Education. 2020;In Press.
12. Schulz KF, Altman DG, Moher D. CONSORT 2010 Statement: updated guidelines for reporting parallel group randomised trials. BMJ. 2010;340.
13. Chan A-W, Tetzlaff JM, Altman DG, Laupacis A, Gøtzsche PC, Krleža-Jerić K, et al. SPIRIT 2013 Statement: Defining Standard Protocol Items for Clinical Trials. Annals of Internal Medicine. 2013;158(3):200-7.
14. James H, Chapman A, Pattison G, Fisher JG, Griffin DR. Assessment of technical skill acquisition and operative competence in Trauma and Orthopaedic surgical training: A systematic review. JBJS(Am) Reviews. 2019.
15. Costa ML, Griffin XL, Achten J, Metcalfe D, Judge A, Pinedo-Villanueva R, et al. World Hip Trauma Evaluation (WHiTE): framework for embedded comprehensive cohort studies. BMJ Open. 2016;6(10):e011679.
16. Hargreaves DH, Bowditch MG, Griffin DR. On-the-job training for surgeons. 1 ed. London: The Royal Society of Medicine Press; 1997.

Author Statement/Contributions

HKJ designed the study and wrote the manuscript

GTRP co-designed the study and the intervention and edited the manuscript

For peer review only

JDF edited the manuscript, made a substantial contribution to the design and is lead supervisor for the qualitative part of the project

DG co-designed the study, edited draft protocols and is lead supervisor for the quantitative part of the project

Acknowledgements

None

For peer review only

Reporting checklist for protocol of a clinical trial.

Based on the SPIRIT guidelines.

Instructions to authors

Complete this checklist by entering the page numbers from your manuscript where readers will find each of the items listed below.

Your article may not currently address all the items on the checklist. Please modify your text to include the missing information. If you are certain that an item does not apply, please write "n/a" and provide a short explanation.

Upload your completed checklist as an extra file when you submit to a journal.

In your methods section, say that you used the SPIRITreporting guidelines, and cite them as:

Chan A-W, Tetzlaff JM, Altman DG, Laupacis A, Gøtzsche PC, Krleža-Jerić K, Hróbjartsson A, Mann H, Dickersin K, Berlin J, Doré C, Parulekar W, Summerskill W, Groves T, Schulz K, Sox H, Rockhold FW, Rennie D, Moher D. SPIRIT 2013 Statement: Defining standard protocol items for clinical trials. Ann Intern Med. 2013;158(3):200-207

For peer review only

		Reporting Item	Page Number
**Administrative information**			
Title	#1	Descriptive title identifying the study design, population, interventions, and, if applicable, trial acronym	1
Trial registration	#2a	Trial identifier and registry name. If not yet registered, name of intended registry	3
Trial registration: data set	#2b	All items from the World Health Organization Trial Registration Data Set	3
Protocol version	#3	Date and version identifier	3
Funding	#4	Sources and types of financial, material, and other support	15
Roles and responsibilities: contributorship	#5a	Names, affiliations, and roles of protocol contributors	1

Roles and responsibilities: sponsor contact information	#5b	Name and contact information for the trial sponsor	15
Roles and responsibilities: sponsor and funder	#5c	Role of study sponsor and funders, if any, in study design; collection, management, analysis, and interpretation of data; writing of the report; and the decision to submit the report for publication, including whether they will have ultimate authority over any of these activities	14
Roles and responsibilities: committees	#5d	Composition, roles, and responsibilities of the coordinating centre, steering committee, endpoint adjudication committee, data management team, and other individuals or groups overseeing the trial, if applicable (see Item 21a for data monitoring committee)	14
**Introduction**			
Background and rationale	#6a	Description of research question and justification for undertaking the trial, including summary of relevant studies (published and unpublished) examining benefits and harms for each intervention	1
Background and rationale: choice of comparators	#6b	Explanation for choice of comparators	11
Objectives	#7	Specific objectives or hypotheses	5-6
Trial design	#8	Description of trial design including type of trial (eg, parallel group, crossover, factorial, single group), allocation ratio, and framework (eg, superiority, equivalence, non-inferiority, exploratory)	5
**Methods: Participants, interventions, and outcomes**			
Study setting	#9	Description of study settings (eg, community clinic, academic hospital) and list of countries where data will be collected. Reference to where list of study sites can be obtained	10

For peer review only

Eligibility criteria	#10	Inclusion and exclusion criteria for participants. If applicable, eligibility criteria for study centres and individuals who will perform the interventions (eg, surgeons, psychotherapists)	9
Interventions: description	#11a	Interventions for each group with sufficient detail to allow replication, including how and when they will be administered	11-13
Interventions: modifications	#11b	Criteria for discontinuing or modifying allocated interventions for a given trial participant (eg, drug dose change in response to harms, participant request, or improving / worsening disease)	N/A – educational trial
Interventions: adherance	#11c	Strategies to improve adherence to intervention protocols, and any procedures for monitoring adherence (eg, drug tablet return; laboratory tests)	N/A – educational trial
Interventions: concomitant care	#11d	Relevant concomitant care and interventions that are permitted or prohibited during the trial	N/A – educational trial
Outcomes	#12	Primary, secondary, and other outcomes, including the specific measurement variable (eg, systolic blood pressure), analysis metric (eg, change from baseline, final value, time to event), method of aggregation (eg, median, proportion), and time point for each outcome. Explanation of the clinical relevance of chosen efficacy and harm outcomes is strongly recommended	6-7
Participant timeline	#13	Time schedule of enrolment, interventions (including any run-ins and washouts), assessments, and visits for participants. A schematic diagram is highly recommended (see Figure)	11
Sample size	#14	Estimated number of participants needed to achieve study objectives and how it was determined, including clinical and statistical assumptions supporting any sample size calculations	6
Recruitment	#15	Strategies for achieving adequate participant enrolment to reach target sample size	8

For peer review only

**Methods: Assignment of interventions (for controlled trials)**			
Allocation: sequence generation	#16a	Method of generating the allocation sequence (eg, computer-generated random numbers), and list of any factors for stratification. To reduce predictability of a random sequence, details of any planned restriction (eg, blocking) should be provided in a separate document that is unavailable to those who enrol participants or assign interventions	10
Allocation concealment mechanism	#16b	Mechanism of implementing the allocation sequence (eg, central telephone; sequentially numbered, opaque, sealed envelopes), describing any steps to conceal the sequence until interventions are assigned	10
Allocation: implementation	#16c	Who will generate the allocation sequence, who will enrol participants, and who will assign participants to interventions	10
Blinding (masking)	#17a	Who will be blinded after assignment to interventions (eg, trial participants, care providers, outcome assessors, data analysts), and how	13
Blinding (masking): emergency unblinding	#17b	If blinded, circumstances under which unblinding is permissible, and procedure for revealing a participant's allocated intervention during the trial	N/A - educational trial
**Methods: Data collection, management, and analysis**			
Data collection plan	#18a	Plans for assessment and collection of outcome, baseline, and other trial data, including any related processes to promote data quality (eg, duplicate measurements, training of assessors) and a description of study instruments (eg, questionnaires, laboratory tests) along with their reliability and validity, if known. Reference to where data collection forms can be found, if not in the protocol	14
Data collection plan: retention	#18b	Plans to promote participant retention and complete follow-up, including list of any outcome data to be collected for	14

For peer review only

		participants who discontinue or deviate from intervention protocols	
Data management	#19	Plans for data entry, coding, security, and storage, including any related processes to promote data quality (eg, double data entry; range checks for data values). Reference to where details of data management procedures can be found, if not in the protocol	14
Statistics: outcomes	#20a	Statistical methods for analysing primary and secondary outcomes. Reference to where other details of the statistical analysis plan can be found, if not in the protocol	14-15
Statistics: additional analyses	#20b	Methods for any additional analyses (eg, subgroup and adjusted analyses)	14-15
Statistics: analysis population and missing data	#20c	Definition of analysis population relating to protocol non-adherence (eg, as randomised analysis), and any statistical methods to handle missing data (eg, multiple imputation)	15
**Methods: Monitoring**			
Data monitoring: formal committee	#21a	Composition of data monitoring committee (DMC); summary of its role and reporting structure; statement of whether it is independent from the sponsor and competing interests; and reference to where further details about its charter can be found, if not in the protocol. Alternatively, an explanation of why a DMC is not needed	14
Data monitoring: interim analysis	#21b	Description of any interim analyses and stopping guidelines, including who will have access to these interim results and make the final decision to terminate the trial	14
Harms	#22	Plans for collecting, assessing, reporting, and managing solicited and spontaneously reported adverse events and other unintended effects of trial interventions or trial conduct	N/A – educational trial
Auditing	#23	Frequency and procedures for auditing trial conduct, if any, and whether the process will be independent from investigators and the sponsor	N/A – educational trial
**Ethics and dissemination**			

For peer review only

Research ethics approval	#24	Plans for seeking research ethics committee / institutional review board (REC / IRB) approval	3
Protocol amendments	#25	Plans for communicating important protocol modifications (eg, changes to eligibility criteria, outcomes, analyses) to relevant parties (eg, investigators, REC / IRBs, trial participants, trial registries, journals, regulators)	N/A – educational trial
Consent or assent	#26a	Who will obtain informed consent or assent from potential trial participants or authorised surrogates, and how (see Item 32)	9
Consent or assent: ancillary studies	#26b	Additional consent provisions for collection and use of participant data and biological specimens in ancillary studies, if applicable	N/A – educational trial
Confidentiality	#27	How personal information about potential and enrolled participants will be collected, shared, and maintained in order to protect confidentiality before, during, and after the trial	14
Declaration of interests	#28	Financial and other competing interests for principal investigators for the overall trial and each study site	16
Data access	#29	Statement of who will have access to the final trial dataset, and disclosure of contractual agreements that limit such access for investigators	14
Ancillary and post trial care	#30	Provisions, if any, for ancillary and post-trial care, and for compensation to those who suffer harm from trial participation	N/A – educational trial
Dissemination policy: trial results	#31a	Plans for investigators and sponsor to communicate trial results to participants, healthcare professionals, the public, and other relevant groups (eg, via publication, reporting in results databases, or other data sharing arrangements), including any publication restrictions	15
Dissemination policy: authorship	#31b	Authorship eligibility guidelines and any intended use of professional writers	15
Dissemination policy: reproducible research	#31c	Plans, if any, for granting public access to the full protocol, participant-level dataset, and statistical code	N/A

Appendices

For peer review only

Informed consent materials	#32	Model consent form and other related documentation given to participants and authorised surrogates	Appendix
Biological specimens	#33	Plans for collection, laboratory evaluation, and storage of biological specimens for genetic or molecular analysis in the current trial and for future use in ancillary studies, if applicable	N/A – educational trial

None The SPIRIT checklist is distributed under the terms of the Creative Commons Attribution License CC-BY-ND 3.0. This checklist can be completed online using https://www.goodreports.org/, a tool made by the EQUATOR Network in collaboration with Penelope.ai

For peer review only
